# 4-Heteroaryl Substituted Amino-3,5-Dicyanopyridines as New Adenosine Receptor Ligands: Novel Insights on Structure-Activity Relationships and Perspectives

**DOI:** 10.3390/ph15040478

**Published:** 2022-04-14

**Authors:** Daniela Catarzi, Flavia Varano, Erica Vigiani, Sara Calenda, Fabrizio Melani, Katia Varani, Fabrizio Vincenzi, Silvia Pasquini, Natascia Mennini, Giulia Nerli, Diego Dal Ben, Rosaria Volpini, Vittoria Colotta

**Affiliations:** 1 Dipartimento di Neuroscienze, Psicologia, Area del Farmaco e Salute del Bambino, Sezione di Farmaceutica e Nutraceutica, Università degli Studi di Firenze, Via Ugo Schiff, 6, 50019 Sesto Fiorentino, Italy; flavia.varano@unifi.it (F.V.); erica.vigiani@unifi.it (E.V.); sara.calenda@unifi.it (S.C.); fabrizio.melani@unifi.it (F.M.); vittoria.colotta@unifi.it (V.C.); 2 Dipartimento di Medicina Traslazionale, Università degli Studi di Ferrara, Via Luigi Borsari 46, 44121 Ferrara, Italy; katia.varani@unife.it (K.V.); fabrizio.vincenzi@unife.it (F.V.); silvia.pasquini@unife.it (S.P.); 3 Dipartimento di Chimica Ugo Schiff, Università degli Studi di Firenze, Via della Lastruccia, 3, 50019 Sesto Fiorentino, Italy; natascia.mennini@unifi.it (N.M.); giulia.nerli@unifi.it (G.N.); 4 Scuola di Scienze del Farmaco e dei Prodotti della Salute, Università degli Studi di Camerino, Via S.Agostino 1, 62032 Camerino, Italy; diego.dalben@unicam.it (D.D.B.); rosaria.volpini@unicam.it (R.V.)

**Keywords:** G-protein-coupled receptors, adenosine receptor ligands, aminopyridine-3,5-dicarbonitriles, ligand-adenosine receptor modeling studies

## Abstract

A new set of amino-3,5-dicyanopyridines was synthesized and biologically evaluated at the adenosine receptors (ARs). This chemical class is particularly versatile, as small structural modifications can influence not only affinity and selectivity, but also the pharmacological profile. Thus, in order to deepen the structure–activity relationships (SARs) of this series, different substituents were evaluated at the diverse positions on the dicyanopyridine scaffold. In general, the herein reported compounds show nanomolar binding affinity and interact better with both the human (h) A_1_ and A_2A_ ARs than with the other subtypes. Docking studies at hAR structure were performed to rationalize the observed affinity data. Of interest are compounds **1** and **5**, which can be considered as pan ligands as binding all the ARs with comparable nanomolar binding affinity (A_1_AR: **1**, *K_i_* = 9.63 nM; **5**, *K_i_* = 2.50 nM; A_2A_AR: **1**, *K_i_* = 21 nM; **5**, *Ki* = 24 nM; A_3_AR: **1**, *Ki* = 52 nM; **5**, *Ki* = 25 nM; A_2B_AR: **1**, EC_50_ = 1.4 nM; **5**, EC_50_ = 1.12 nM). Moreover, these compounds showed a partial agonist profile at all the ARs. This combined AR partial agonist activity could lead us to hypothesize a potential effect in the repair process of damaged tissue that would be beneficial in both wound healing and remodeling.

## 1. Introduction

The amino-3,5-dicyanopyridines have attracted much attention due to their versatility to behave as AR ligands. In fact, they are endowed with not only a wide range of affinity but also with different degrees of activities, with their profile varying from full to partial agonist or neutral antagonist at the different ARs [1,2,3,4,5,6,7]. As for other G-protein-coupled receptors [8,9,10], some of the antagonists have been proven to be inverse agonists [6]. The interest in modulating the effects of the natural ligand adenosine ensued from the evidence of its involvement in a large variety of physiological functions throughout the body. Interaction of the ubiquitous adenosine with its four G-protein-coupled A_1_, A_2A_, A_2B_ and A_3_ ARs produced different responses depending on the type of AR and consequent cellular signaling involved. In fact, the A_1_ and A_3_ ARs are coupled to G_i_ proteins, which inhibit adenylate cyclase (AC), whereas A_2A_ and A_2B_ receptors signal through G_s_ proteins, thus activating it. Coupling to other second messenger systems has also been described [11,12].

Under stress conditions, extracellular adenosine grow from physiological nanomolar concentrations (30–200 nM) to high micromolar levels (30 µM), thus activating the diverse AR subtypes [12].

Adenosine regulates neurotransmitter release [13], synaptic plasticity [14] and neuroprotection in the central nervous system (CNS) [12,15,16] and plays different roles in a large variety of tissues and organs. In particular, adenosine controls T-cell proliferation and cytokine production [17] and produces either vasoconstriction or vasodilation [18] in the cardiovascular system. The nucleoside also inhibits lipolysis and stimulates bronchoconstriction [19,20].

Growing interest is emerging for its involvement also in wound healing and remodeling processes, including different stages, such as inflammation, neovascularization and tissue regeneration [21,22]. In fact, skin-lesion-associated diseases are one of the most common afflictions in the world, but their incidence on human health is still underestimated [23]. It is well-known that all ARs are involved, but their role has not yet been fully clarified [22,24]. AR agonists were reported to promote wound healing at different levels of the tissue-repair process, depending on the subtype activated. Thus, the use of mixed or pan agonists capable of simultaneously activating more receptor subtypes could lead to a synergistic effect [24,25].

Many publications report the amino-3,5-dicyanopyridines as AR ligands, showing nanomolar affinity and ranging from pan to selective AR ligands [1,2,3,4,5,6,7]. Moreover, this series seems to be more eclectic for pharmacological studies, because it is endowed with less species’ differences with respect to the adenosine-like AR agonists [26].

**Figure 1:**
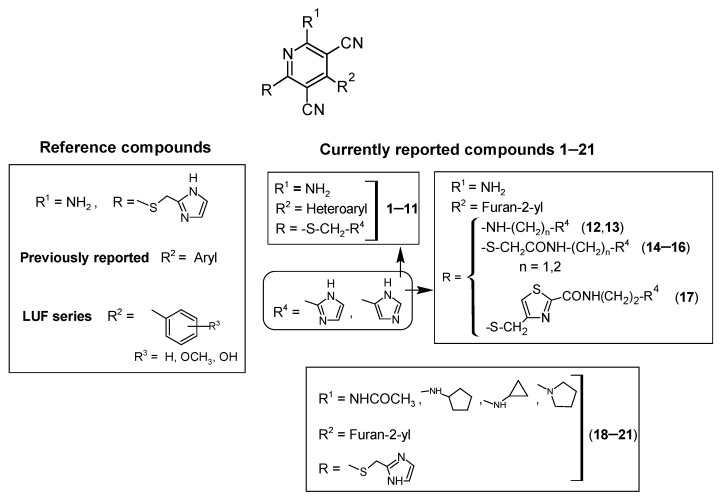
Lead structures and currently reported amino-3,5-dicyanopyridine-based adenosine receptor ligands.

In recent years, we have produced a lot of AR ligands belonging to the amino-3,5-dicyanopyridine series [5,6,7]. In particular, in the last publication [7], a set of 4-aryl substituted dicyanopyridines bearing a *1H*-imidazol-2-ylmethylsulfanyl group at R position, as in the parent LUF series [2], was described (Figure 1). The previously reported study demonstrated the key role of the R^2^ substituent in addressing affinity and selectivity toward specific ARs [6]. Thus, in order to deepening the SARs at this level, different heteroaryl moieties were introduced at R^2^, while maintaining the methylsulfanyl linker between the dicyanopyridine scaffold and the “usual” 1*H*-imidazol-2-yl group (compounds **1**–**10**) or the “new entry” *1H*-imidazol-4-yl moiety (derivative **11**). Moreover, keeping constant the furan-2-yl moiety at 4-position, the R substituent was modified by varying the nature and the length of the linker bearing the imidazole as the terminal group (compounds **12**–**17**). In the meanwhile, the primary amine function (R^1^) was replaced by different secondary or tertiary amino-substituents (compounds **18**–**20**), or acetylated (**21**).

## 2. Results

### 2.1. Chemistry

**Scheme 1:**
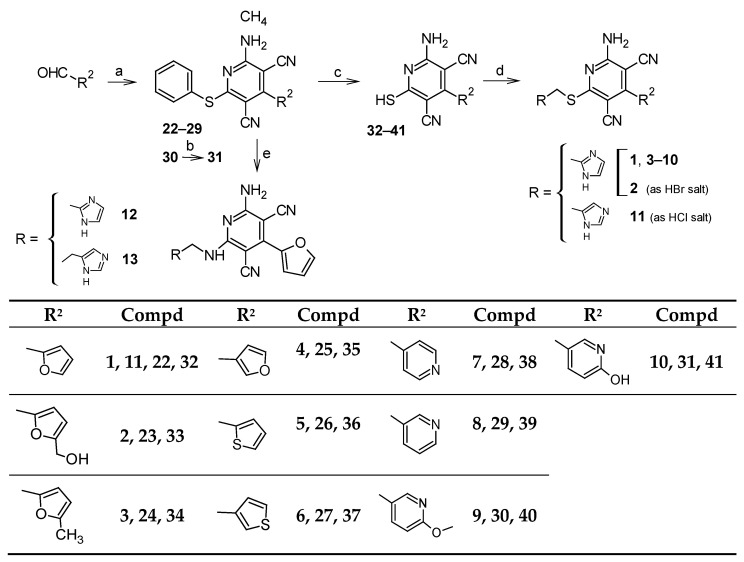
Reagents and conditions: (**a**) malononitrile, thiophenol, TBAF·H_2_O, H_2_O, 80 °C (compounds **22**–**27**, **29**, **30**); malononitrile, thiophenol, basic Al_2_O_3_, H_2_O, 100 °C (compound **28**); (**b**) HBr 33% solution in CH_3_COOH, 70 °C; (**c**) Na_2_S, anhydrous DMF, 80 °C; 1N HCl, RT; (**d**) 2-bromomethyl)-1-H-imidazole hydrobromide (for compounds **1**–**10**) or 4-(chloromethyl)-1*H*-imidazole hydrochloride (**53**) (for compound **11**), NaHCO_3_, anhydrous DMF, RT (see experimental for details); (**e**) anhydrous Et_3_N, anhydrous DMF, RCH_2_NH_2_ (1-(1*H*-imidazol-2-yl)methanamine hydrochloride for compound **12**, or 2-(1*H*-imidazol-4-yl)ethanamine for **13**), MW, 120 °C.

**Scheme 2:**
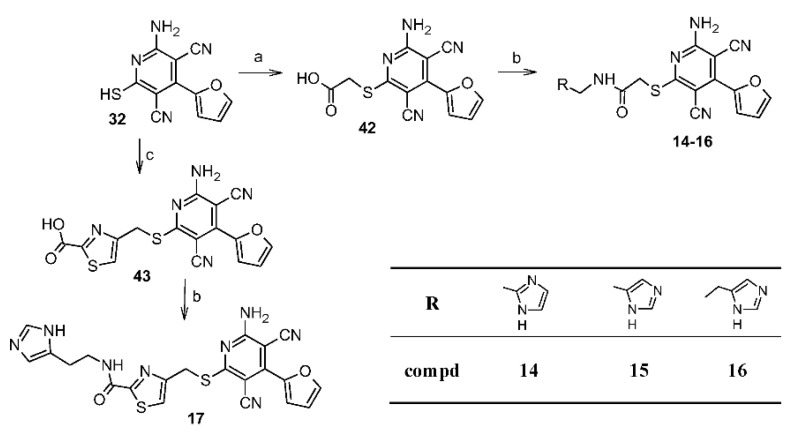
Reagents and conditions: (**a**) ClCH_2_COOH, NaHCO_3_, anhydrous DMF, RT, N_2_ atmosphere; H_2_O, 6 M HCl (pH 2); (**b**) R-CH_2_-NH_2_ (1-(1*H*-imidazol-2-yl)methanamine hydrocloride, for compound **14**; or 1-(1*H*-imidazol-4-yl)methanamine (**55**), for compound **15**); or 2-(1*H*-imidazol-4-yl)ethanamine, for compounds **16**, **17**), 1-ethyl-3-(3-dimethylaminopropyl)carbodiimide hydrochloride, 1-hydroxybenzotriazole hydrate, Et_3_N, anhydrous DMF, RT; (**c**) 4-(chloromethyl)-1,3-thiazole-2-carboxylic acid (**57**), NaHCO_3_, anhydrous DMF, RT.

**Scheme 3:**
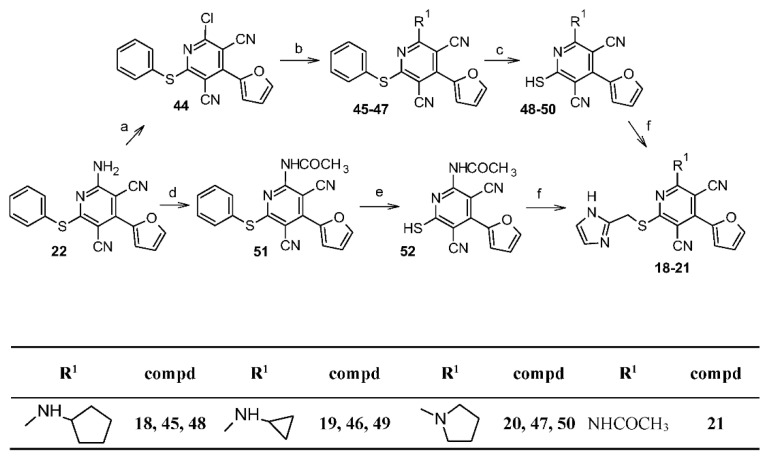
Reagents and conditions: (**a**) isoamyl nitrite, CuCl_2_, anhydrous CH_3_CN, RT; (**b**) R^1^-H, anhydrous DMF, RT; (**c**) Na_2_S, anhydrous DMF, 80 °C; 1 N HCl, RT; (**d**) acetic anhydride, anhydrous pyridine, reflux; (**e**) Na_2_S, anhydrous DMF, 50 °C; 1 N HCl, RT; (**f**) 2-(bromomethyl)-1*H*-imidazole hydrobromide, NaHCO_3_, anhydrous DMF, RT.

**Scheme 4:**
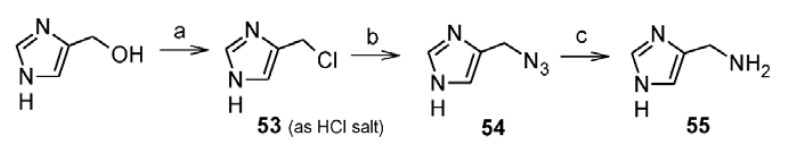
Reagents and conditions: (**a**) SOCl_2_, reflux; (**b**) NaN_3_, EtOH, DMF (catalytic amount), 70 °C; (**c**) H_2_ (1 Atm), 10% Pd/C, EtOH.

**Scheme 5:**
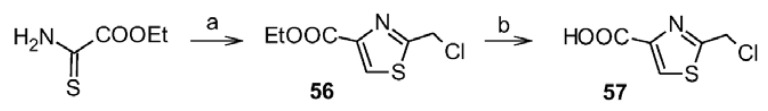
Reagents and conditions: (**a**) 1,3-dichloroacetone, anhydrous acetone, reflux; (**b**) LiOH, 1:1 CH_3_CN/H_2_O, RT; citric acid, RT.

The syntheses of the target compounds **1**–**21** and their intermediates followed the procedures as delineated in Scheme 1, Scheme 2, Scheme 3, Scheme 4 and Scheme 5. Derivatives **1**–**10** were prepared starting from the 6-sulfanyl-substituted compounds **32**–**41** [6,27,28,29], which were synthesized as depicted in Scheme 1. The suitable heteroaryl aldehydes were reacted with malononitrile and thiophenol in the presence of tetrabuthylammonium fluoride hydrate (for compounds **22**–**27**, **29** [6,30,31,32] and **30**) or basic alumina (for **28**) [31,33] to yield the suitable 6-phenylsulfanyl-derivatives **22**–**30** [6,30,31,32,33]. Compound **31**, bearing at 4-position a 5-pyridin-2-ol moiety, was obtained from the corresponding 2-methoxy derivative **30** by treatment with hydrobromic acid (33% in glacial acetic acid), at 70 °C. When **22**–**31** [6,30,31,32,33] were treated with anhydrous sodium sulfide in anhydrous DMF at 80 °C, followed by acidification with 1N HCl, the free thiols **32**–**41** [6,27,28,29] were obtained in high yields. Reaction of **32**–**34** [6,27] with equimolar amount of 2-bromomethyl-*1H*-imidazole hydrobromide [5] gave the hydrobromide salt of the target compounds **1**–**3.** While compound **2** was isolated and characterized as such, derivatives **1** and **3** were obtained by treatment of the corresponding hydrobromide salt with sodium hydrogen carbonate at room temperature. Compounds **4**–**10** were obtained starting from the suitable sulfanyl derivatives **35**–**41** [6,27,28,29]. Compound **11** was obtained by reacting the sulfanyl derivative **32** [6,27] with 4-chloromethyl-*1H*-imidazole hydrochloride **53** [34] (see Scheme 4 below), while the 2-amino-substituted derivatives **12** and **13** were generated by the microwave-assisted reaction between the phenylsulfanyl compound **22** and the commercially available (1-(1*H*-imidazol-2-yl)methanamine hydrochloride or 2-(1*H*-imidazol-4-yl)ethanamine, respectively, in DMF, at 120 °C, in the presence of Et_3_N. 

The synthesis of the target dicyanopyridines **14**–**17** starting from the common intermediate **32** is reported in Scheme 2. Treatment of the latter with chloroacetic acid or 4-(chloromethyl)-1,3-thiazole-2-carboxylic acid (**57**) [35] (see Scheme 5 below) in anhydrous DMF, in mild alkaline conditions (NaHCO_3_), furnished the 2-sulfanyl-acetic acid **42** and the 2-methylsulfanyl-1,3-thiazole-2-carboxylic acid derivative **43**, respectively. Compound **42** was isolated from the crude mass reaction by acidification with 6M HCl. Both **42** and **43** were converted respectively into the target derivatives **14**–**16** and **17**, with variable yields, by reacting with (1-(1*H*-imidazol-2-yl)methanamine hydrochloride (compound **14**), 1-(1*H*-imidazol-4-yl)methanamine (**55**) (see Scheme 4 below) [36] (compound **15**) or 2-(1*H*-imidazol-4-yl)ethanamine (compounds **16** and **17**), in anhydrous DMF, in the presence of 1-ethyl-3-(3-dimethylaminopropyl)carbodiimide hydrochloride, 1-hydroxybenzotriazole hydrate and Et_3_N. 

Diazotization of the phenylsulfanyl-derivative **22** [30] with isoamyl nitrite in anhydrous CH_3_CN, followed by treatment with CuCl_2_, yielded 2-chloro-3,5-dicyanopyridine **44**, which was reacted with the suitable amine in anhydrous DMF at RT to give compounds **45**–**47** (Scheme 3). Reaction of the latter with sodium sulfide in anhydrous DMF at 80 °C furnished the 2(6)-sulfanyl-substituted pyridine derivatives **48**–**50** as key intermediates to obtain the target compounds **18**–**20**. The reaction of **22** [30] with acetic anhydride in anhydrous pyridine at reflux gave 6-phenylsulfanyl-2-acetamide **51** [5], which was treated with anhydrous sodium sulfide in anhydrous DMF, at 50 °C, followed by 1N HCl, to yield the corresponding 6-sulfanyl derivative **52** [5]. The target compounds **18**–**21** were obtained by treatment of **48**–**50** and **52** [5] with 2-(bromomethyl)-1*H*-imidazole hydrobromide [5] in anhydrous DMF, in mild alkaline conditions. 

The not commercially available reactants (**53** [34], **55** [36] and **57** [35]) that are useful to synthesize compounds **11**, **15** and **43** were prepared as depicted in Scheme 4 and Scheme 5. Treatment of the commercially available 1*H*-imidazol-4-ylmethanol with an excess of thionyl chloride at reflux gave 4-(chloromethyl)-1*H*-imidazole (**53**) [34], which was converted into the 4-(azidomethyl) intermediate **54** by reacting with sodium azide in EtOH in the presence of a catalytic amount of DMF, at 70 °C (Scheme 4). Hydrogenation of the latter in a Parr apparatus (1 atm), with 10% Pd/C as catalyst in EtOH, yielded the 1-(1*H*-imidazol-4-yl)methanamine (**55**) [36] in high yield. Reaction of ethyl thiooxamate and 1,3-dichloroacetone in anhydrous acetone at reflux gave ethyl 4-(chloromethyl)thiazole-2-carboxylate **56** [35], which was hydrolyzed with lithium hydroxide in aqueous (1:1) CH_3_CN, followed by acidification with citric acid, to furnish the corresponding carboxylic acid **57** [35] (Scheme 5).

The analysis of the ^1^H-NMR spectra of the target amino-3,5-dicyanopyridines, bearing a 2-(1*H*-imidazol-2-yl-methyl)sulphanyl substituent on the 6-side chain (**1**–**10**, **12**, **18**–**21**), featured a particular behavior. In the ^1^H NMR spectra of both the crude and the purified compounds (**1**–**3, 5, 8**, **9** and **18**–**21)**, the two imidazole protons at the 4 and 5 position appeared as a single signal (singlet) around 7 ppm, which integrated 2 (equivalent protons), while the NH proton was a very broad signal from 12 to 13 ppm. The same applied for compounds **4**, **6**, **7**, **10** and **12** before purification. In this situation, it was possible to hypothesize a fast tautomeric equilibrium, which determines the equivalence of the two protons and influences the relaxation time of the imidazole NH group (broad signal). However, the pattern of the ^1^H NMR spectra of compounds **4**, **6**, **7**, **10** and **12** changed after crystallization or purification by silica gel column chromatography. In fact, the two imidazole protons at 4,5 appeared as distinct signals falling around 6.8 and 7.1 ppm, while the signal of the NH proton was a singlet around 12 ppm. This experimental evidence led us to hypothesize that the two distinct signals of the imidazole protons in the purified compounds **4**, **6**, **7**, **10** and **12** indicated the formation of an intramolecular hydrogen bond between the pyridine nitrogen at position 1 and the imidazole NH on the 6-side chain. The two distinct signals could be due to the stiffening of the sulfanylmethyl–imidazole chain as a result of which the neighborhood of the two protons at 4 and 5 becomes different. This hypothesis was supported by a ^1^H NMR study of compound **10**, taken as reference. A detailed report of the registered spectra is reported in the Supplementary Materials. Moreover, an ab initio quantum mechanical calculation on compound **10**, and also on the other congeners, namely **4**, **6**, **7** and **12**, was performed. The program used was GAMESS (General Atomic and Molecular Electronic Structure System) [37], and the discussion is reported in the Section 3.2 (“Ab Initio Quantum Mechanical Studies”).

### 2.2. Pharmacological Assays

The amino-3,5-dicyanopyridines **1**–**21** were tested for their affinity at hA_1_, hA_2A_ and hA_3_ ARs, stably transfected in Chinese Hamster Ovary (CHO) cells. Moreover, they were also studied as hA_2B_ agonists by evaluating their stimulatory effect on cAMP production in CHO cells, stably expressing the hA_2B_ AR. Compounds **1**, **5**, **16** and **17**, the most interesting in terms of affinity at the hA_1_AR, were evaluated for their efficacy at the hA_1_ receptor. Due to their interesting pan profile, both compounds **1** and **5** were evaluated as AR agonists also at the hA_2A_ and hA_3_ subtypes. Each compound was tested in the cAMP assay to assess its capability to modulate Forskolin-stimulated cAMP levels in the absence and/or presence of 2-chloro-*N*^6^-cyclopentyladenosine (CCPA, as an A_1_AR full agonist, set at 100%); 8-cyclopentyl-1,3-dipropylxanthine (DPCPX as an A_1_AR inverse agonist set at −100%); 2-*p*-(2-carboxyethyl)phenethylamino-5′-*N*-ethylcarboxamidoadenosine (CGS21680 as an A_2A_AR full agonist set at 100%); 5-[2-chloro-6-[(3-iodophenyl)methylamino]purin-9-yl]-3,4-dihydroxy-*N*-methyloxolane-2-carboxamide (2-Cl-IB-MECA as an A_3_AR full agonist set at 100%). All pharmacological data are reported in Table 1 and Table 2. 

### 2.3. Molecular Docking Studies 

Molecular docking studies were performed at the hA_1_AR and hA_2A_AR 3D structures to analyze the binding data of the synthesized compounds. The cryo-EM structure of the adenosine-bound hA_1_AR (pdb code: 6D9H; 3.6-Å resolution [38]) and the crystal structure of the hA_2A_R in complex with the inverse agonist ZM241385 (pdb code: 4EIY; 1.8-Å resolution [39]) were retrieved from the protein data bank (https://www.rcsb.org/, accessed on 6 April 2012) and employed as molecular targets. Docking analyses were performed with the CCDC Gold [40] docking algorithm by using MOE (Molecular Operating Environment, version 2019.0101) suite [41] as the interface.

### 2.4. In Vitro Permeation Studies

Compounds **1** and **5** were evaluated for their capability to penetrate the artificial membrane simulating the epidermal barrier. Permeation flux was assessed by using vertical Franz diffusion cells [42].

## 3. Discussion

### 3.1. Structure–Activity Relationships

The pharmacological results of the newly synthesized amino-3,5-dicyanopyridine derivatives **1**–**21** and those of the reference compound LUF5833 (2-amino-6-[(1*H*-imidazol-2-ylmethyl)sulfanyl]-4-phenylpyridine-3,5-dicarbonitrile) [2] are reported in Table 1 and Table 2. To better follow the discussion in both this section and in the molecular modeling one, LUF5833 was considered as a reference to define the numbering of the dicyanopyridine core to refer to. The pyridine nitrogen atom represents position 1, while the amino and the sulfanyl function occupy position 2 and 6, respectively. 

Most of the reported compounds were devoid of an affinity for the hA_2B_AR, with the exception of derivatives **1**, **4** and **5**–**8** showing EC_50_ values for this receptor below 63 nM (Table 1). As observed for other reported set of this series [5,6,7], the A_3_AR affinity was, in general, null or fell in the micromolar range. The only two exceptions were compounds **1** and **5**, which displayed a nanomolar K_i_ value for this receptor subtype. In general, the herein reported compounds interacted better with both the hA_1_ and A_2A_ ARs than with the other subtypes. However, compounds **1** and **5**, which can be considered as pan ligands binding all the ARs with comparable affinity and in the nanomolar range, were interesting. The analogs **4** and **6** had a similar trend though binding the hA_3_AR with lower affinity with respect to **1** and **5** but similarly to the lead LUF5833. Moreover, compounds **1** and **5,** when evaluated in the functional tests, showed a partial agonist profile at all the ARs (Table 2). 

**Table 1:** Binding affinities (*K_i_*) at hA_1_, hA_2A_ and hA_3_ ARs and potencies (EC_50_) at hA_2B_ ARs.

	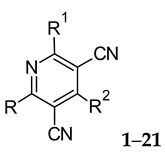						
				Binding Experiments ^a^			cAMP Assays
				*K*_i_ (nM) or I%			EC_50_ (nM) ^b^ Efficacy ^c^ (%)
Compd	R^1^	R^2^	R	hA_1_^d^	hA_2A_^e^	hA_3_^f^	hA_2B_
**1**	NH_2_	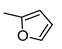	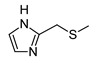	9.63 ± 1.61	21 ± 2	52 ± 5	1.4 ± 0.2 (52%)
**2 ^g^**	NH_2_	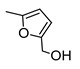	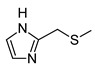	190 ± 16	233 ± 19	290 ± 23	>1000 (8%)
**3**	NH_2_	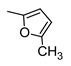	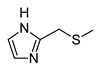	10.1 ± 0.8	10.5 ± 0.9	279 ± 21	>1000 1%
**4**	NH_2_	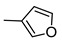	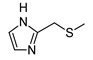	0.77 ± 0.09	37 ± 3	274 ± 23	2.32 ± 0.21 (42%)
**5**	NH_2_	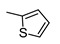	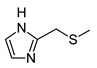	2.50 ± 0.20	24 ± 2	25 ± 2	1.12 ± 0.11 (73%)
**6**	NH_2_	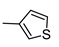	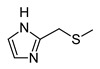	1.01 ± 0.09	55 ± 6	221 ± 20	3.15 ± 0.29 (47%)
**7**	NH_2_	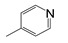	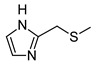	8.85 ± 0.82	81 ± 7	26%	12.5 ± 1.3 (36%)
**8**	NH_2_	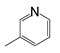	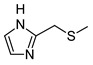	5.26 ± 0.48	463 ± 38	19%	63 ± 5 (58%)
**9**	NH_2_	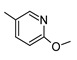	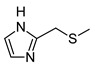	2.71 ± 0.18	377 ± 32	798 ± 72	>1000 (7%)
**10**	NH_2_	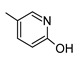	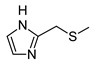	149 ± 11	613 ± 53	23%	>1000 (1%)
**11 ^h^**	NH_2_	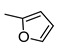	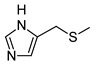	51 ± 4	442 ± 37	849 ± 74	>1000 (4%)
**12**	NH_2_	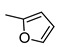	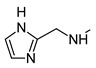	315 ±28	157 ± 14	16%	>1000 (3%)
**13**	NH_2_	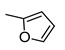	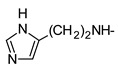	174 ± 14	125 ± 10	6%	>1000 (9%)
**14**	NH_2_	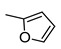	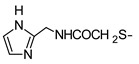	87 ± 7	817 ± 71	33%	>1000 (6%)
**15**	NH_2_	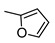	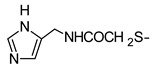	378 ± 26	225 ± 18	758 ± 66	>1000 (1%)
**16**	NH_2_	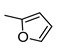	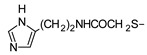	30 ± 3	138 ± 11	30%	>1000 (1%)
**17**	NH_2_	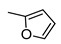	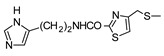	33 ± 3	279 ± 22	542 ± 48	>1000 6%
**18**	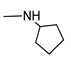	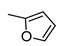	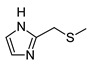	393 ± 32	138 ± 11	279 ± 21	>1000 2%
**19**	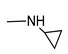	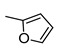	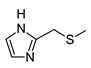	292 ± 26	1%	18%	>1000 (13%)
**20**	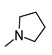	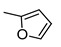	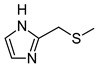	41 ± 3	89 ± 7	118 ± 11	>1000 (8%)
**21**	NHCOCH_3_	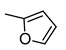	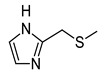	34 ± 3	394 ± 27	768 ± 63	>1000 (1%)
**LUF5833** **^i^**	NH_2_	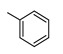	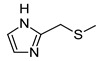	2.4 ± 1	28 ± 1	171 ± 109	19 ± 7 (81%)

^a^*K*_i_ values are means ± SEM of four separate assays each performed in triplicate. Percentage of inhibition (I%) was determined at 1 µM concentration of the tested compounds. ^b^ EC_50_ values are means ± SEM of four separate assays each performed in triplicate. ^c^ Efficacy of the tested compound at 1 µM concentration, in comparison with NECA (1 µM = 100%). ^d^ Displacement of specific [^3^H]DPCPX competition binding to hA_1_CHO cells. ^e^ Displacement of specific [^3^H]ZM241385 competition binding to hA_2A_CHO cells. ^f^ Displacement of specific [^125^I]AB-MECA competition binding to hA_3_CHO cells. ^g^ As hydrobromide salt. ^h^ As hydrochloride salt. ^i^ Reference [2].

**Table 2:** Modulation of Forskolin-stimulated cAMP levels of selected amino-3,5-dicyanopyridine derivatives on cyclic cAMP assay in hA_1_, hA_2A_ and hA_3_ CHO cells ^a^.

	hA_1_AR		hA_2A_AR		hA_3_AR	
Compd	Efficacy, ^b^ % (Profile)	EC_50_ or IC_50_ (nM)	Efficacy, ^b^ %	EC_50_ (nM)	Efficacy, ^b^ %	IC_50_ (nM) ^c^
**1**	31 ± 3 (Partial Agonist)	12.2 ± 1.2 ^c^	39 ± 3	15.1 ± 1.3	45 ± 4	68 ± 6
**5**	75 ± 5 (Partial Agonist)	1.95 ± 0.16 ^c^	40 ± 3	11.3 ± 1.0	34 ± 3	33 ± 2
**11**	0.82 ± 0.07 (Antagonist)	125 ± 11 ^d^	NT ^e^	NT	NT	NT
**12**	1.31 ± 0.11 (Antagonist)	768 ± 67 ^d^	NT	NT	NT	NT
**16**	−43 ± 4 (Inverse Agonist)	59 ± 4 ^c^	NT	NT	NT	NT
**17**	54 ± 4 (Partial Agonist)	38 ± 3 ^c^	NT	NT	NT	NT

^a^ Potency values (EC_50_ or IC_50_) are expressed as means ± SEM of four independent cAMP experiments, each performed in triplicate. ^b^ Efficacy of the novel compounds was normalized by using the efficacy value of the reference compounds: CCPA as A_1_AR full agonist (set at 100%); DPCPX as A_1_AR inverse agonist (set at −100%); CGS21680 as A_2A_AR full agonist (set at 100%); 2-Cl-IB-MECA as A_3_AR full agonist (set at 100%). ^c^ Potency of the novel compounds to modulate Forskolin-stimulated cAMP levels. ^d^ Potency of the novel compounds to inhibit the effect of CCPA 10 nM. ^e^ NT = not tested.

In general, keeping constant both the 2-amino function and the 6-(1*H*-imidazol-2-ylmethyl)sulfanyl side chain on the dicyanopyridine core, we can see that the introduction of different heteroaryl groups at the 4 position (compounds **1**–**10**) influenced the binding affinity at the diverse ARs differently. All of these compounds showed an hA_1_AR *K_i_* value below 10 nM, with the only two exceptions being compounds **2** and **10**. A common feature of these latter two compounds was a hydrophilic hydroxyl group appended on the 4-heteroaryl moiety. Regarding the A_2A_ receptor, the presence at the 4-position of either furanyl or thienyl rings (compounds **1–6**) seemed to better promote the binding interaction with this subtype than a pyridine substituent (derivatives **7**–**10**). Moreover, as observed in previously reported set of compounds of this series [5,6,7], the presence of a substituent on the 4-(hetero)aryl moiety dramatically influenced the hA_2B_ AR activity (compare compound **1** to **2**, **3**, and compound **8** to **9**, **10**, respectively). 

As previously reported [6], replacement of the methylsulfanyl linker with a methylamino one led to a high decrease of affinity at all the ARs (compare compound **1** to **12**). Moreover, the presence of a longer 6-linker between the 1*H*-imidazol-2yl group and the dicyanopyridine scaffold in general negatively influenced the affinity at all the ARs, and, to a minor extent, the binding at the A_1_ subtype. In fact, compounds **16** and **17** maintained *K_i_* values in the low nanomolar range. 

The replacement of the methylsulfanyl linker on the 6-side-chain with a methylamino (compound **1** versus **12**), or with a longer one (compound **1** versus **16**), produced a shift of the pharmacological profile from partial agonist at the A_1_AR to antagonist or inverse agonist, respectively. Similarly, when the 1*H*-imidazol-2-yl was replaced with the 1*H*-imidazol-4-yl moiety (compound **1** versus **11**) a change from partial agonist to antagonist profile was observed (Table 2). These data confirm that the requirements at the R position are very precise. In support of this, the inverse agonist profile (compound **16**) was changed back to partial agonist (compound **17**) when the 1,3-thiazol-5-yl moiety was directly attached to the methylsulfanyl linker. This result was in accordance with the literature data [4,43,44,45]. In fact, many amino-3,5-dicyanopyridines bearing the thiazole feature in this precise position were reported as potent and selective A_1_AR agonists.

The introduction of cycloalkyl substituents on the 2-amino function (compounds **18** and **19**) and the inclusion of the latter in a (pyrrolidin-1-yl) moiety (compound **20**) or its acetylation (compound **21**) differently affected the AR binding affinities with respect to the unsubstituted derivative **1**. Interaction with the A_2B_ receptor was completely lost, while affinity was retained at the other AR subtypes. In particular, while compound **20** maintained A_1_ and A_2A_ AR *K_i_* values in the nanomolar range, the 2-acetylamino derivative **21** bound the A_1_ subtype with good affinity.

### 3.2. Ab Initio Quantum Mechanical Studies

**Figure 2:**
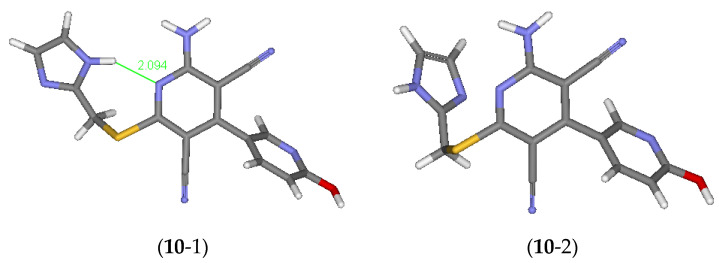
Minimized conformations 1 and 2 of compound **10**.

The hypothesis of the intramolecular H-bond formation between the pyridine nitrogen at position 1 and the imidazole NH hydrogen was supported by ab initio quantum mechanical calculations performed on compounds **4**, **6**, **7**, **10** and **12**. The program used was GAMESS [37]. The formation energies (E_1_ and E_2_) of two conformations, 1 and 2, were calculated (Table 3) with Geometry Optimization, i.e., with conformational optimization. Conformation 1 included the formation of an intramolecular H-bond between the imidazole NH hydrogen and the pyridine nitrogen at position 1. As an example, the two minimized conformations, 1 and 2, of the reference compound **10** (**10**-1 and **10**-2) were depicted in Figure 2. Referring to the compounds under study, negative ΔE values (Table 3) indicated that conformations 1 were slightly more stable than the 2 ones, where the H-bond was not supposed. Thus, the observed energy gain was attributable to the intramolecular H-bond formation, but its low value, which was generally observed, indicated that this hypothetical H-bond was very weak. In fact, in the presence of water, a perturbation of the system, was observed, and the intramolecular H-bond was not formed. In this situation, the imidazole NH hydrogen established a stabilizing H-bond with a water molecule, leading to an energy gain of about −11 Kcal/mol. 

### 3.3. Molecular Modeling Studies 

**Figure 3:**
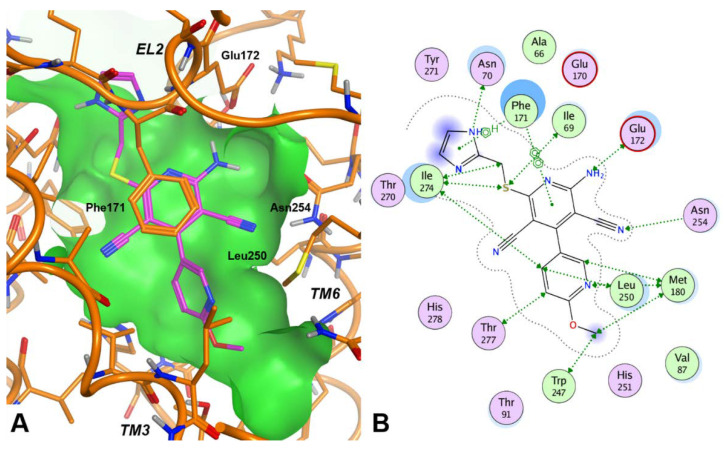
(**A**) Binding mode of the synthesized compounds at the hA_1_AR cavity; **9**-receptor interaction is shown, with indication of key receptor residues. (**B**) Schematic view of the ligand–target interaction (developed with the Ligand Interaction tool within MOE).

Analogously to previously reported dicyanopyridines [5,6,7,46,47], the best-score docking conformations generally observed for the newly synthesized compounds at the hA_1_AR present the pyridine scaffold being inserted between hydrophobic residues conserved among the human ARs (Phe171, Leu250^6.51^ and Ile274^7.39^) and making non-polar interaction with these amino acids (Figure 3 reports the binding mode of **9** within the hA_1_AR cavity). The 2-amino function of **9** makes a double polar interaction with Asn254^6.55^ and Glu172. The 3-cyano also binds Asn254^6.55^ through a polar interaction, while the 5-cyano moiety points toward the depth of the binding cavity positioning itself close to Ala66^2.61^, Ile69^2.64^, Val87^3.32^ and His278^7.43^.

**Figure 4:**
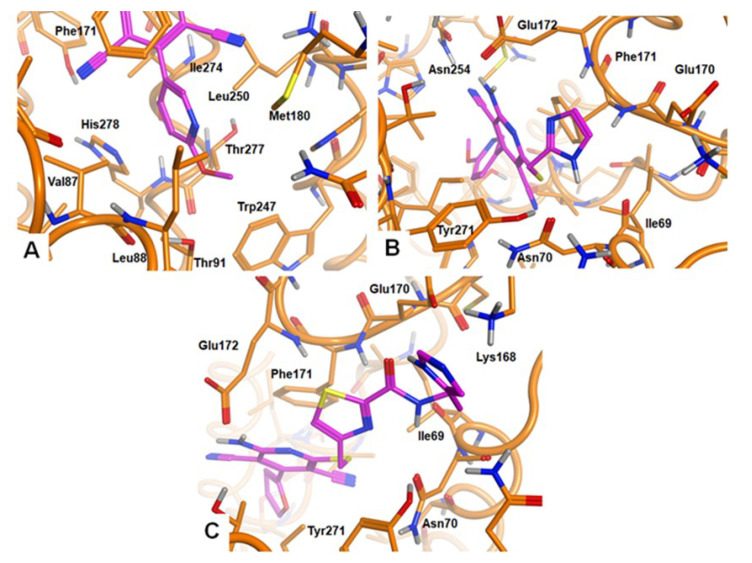
(**A**,**B**) Detailed view of the binding mode of compound **9** within the hA_1_AR cavity. (**A**) Detail of the interaction of the 4-heteroaryl substituent with the receptor residues in proximity. (**B**) Top view of the docking pose of **9** within the hA_1_AR cavity, with indication of the receptor residues in proximity of the 2-substituent. (**C**) Top view of the docking pose of derivative **17** within the hA_1_AR cavity, with indication of the receptor residues in proximity of the 6-side-chain.

The 4-heterocyclic substituent is inserted in the depth of the cavity, close to residues belonging to TM3, EL2, TM5, TM6 and TM7 domains (Val87^3.32^, Leu88^3.33^, Thr91^3.36^, Phe171, Met180^5.38^, Trp247^6.48^, Leu250^6.51^, Ile274^7.39^, Thr277^7.42^ and His278^7.43^). In general, all the AR non-nucleoside agonists reported to date bear an aromatic ring at this position. The affinity data of the compounds synthesized in this work confirm this feature, since the presence of aromatic heterocyclic groups (R^2^) at the 4-position affects affinity, especially for both the A_1_ and the A_2B_ subtypes. In particular, as previously observed [2,7], the introduction of a substituent in the para-position of the 4-aromatic ring modulates the binding affinity. In fact, the presence of substituents on the pyridyl moiety (compounds **9** and **10**) in a position corresponding to the para one in a phenyl ring, or at the 5-position of a 2-furanyl ring (**2** and **3**), modulates the affinity for the AR subtypes compared to the unsubstituted analogues **1** and **8.** Figure 3A and Figure 4A show the interaction of the 2-methoxy-5-pyridyl substituent at 4-position of **9** with the receptor residues. The presence of the additional methoxy group allows the compound to completely fill the narrow sub-cavity in the depth of the binding site (Figure 3A). Since the amino acid residues forming such a sub-cavity are mainly hydrophobic, it is no surprise that the hA_1_AR affinity data of **9** are comparable to those of **8**. When the methoxy group is replaced by a polar hydroxyl function, the affinity gets significantly lower (compound **10**). Analogously, the presence of an additional 5-methyl group on a 4-(2-furanyl) substituent maintains high affinity for the hA_1_AR (derivative **3**) compared to the corresponding unsubstituted analogue **1**. Replacement of the methyl group with a polar hydroxymethyl function (**2**) leads to a decrease of the hA_1_AR affinity.

Similar to what was observed in previously reported docking studies [5,6,7], the 6-substituent points toward the entrance of the binding cavity (Figure 3). As already observed [6], a methylsulfanyl-linker on the 6-side-chain leads to a higher affinity with respect to the aminomethyl one (compare **1** to **12**), probably due to the lack of conjugation effects of the methylsulfanyl-linker (existing instead for the aminomethyl one) that allows this structural feature to better accommodate in the binding site, assuming a non-coplanar position with respect to the dicyanopyridine scaffold. The 1*H*-imidazol-2yl moiety on the 6-side-chain gets positioned between residues belonging to TM2, EL2 and TM7 domains (Ile69^2.64^, Asn70^2.65^, Glu170, Phe171, Glu172, Tyr271^7.36^ and Ile274^7.39^). The location of this substituent, with respect to the receptor residues, modulates, to some extent, the binding at the A_1_AR. In fact, the presence of a longer 4-linker between this group and the dicyanopyridine scaffold generally leads to a slight decrease of affinity at this subtype with respect to the parent compound **1** (see derivatives **14**–**17**). In this sense, docking results show that, for compounds bearing a longer 4-linker, the imidazolyl moiety gets located in a more external position, leading to a different interaction with the receptor residues. The presence of a 4-methylsulfanyl linker and an imidazole-2yl group (compounds **1** and **5**) on the 6-side-chain seems to be correlated to an agonist/partial agonist profile. The replacement of these features respectively with an aminomethyl linker (**12**) or an imidazole-4-yl moiety (**11**) causes a shift to antagonist behavior. The docking results do not explain the observed agonist-to-antagonist activity shift as well as the change from an inverse agonist (compound **16**) to partial agonist profile when a 1,3-thiazole moiety was included into the 6-side-chain (compound **17**). Figure 4C is a top view of the docking pose of **17** within the hA_1_ receptor cavity.

**Figure 5:**
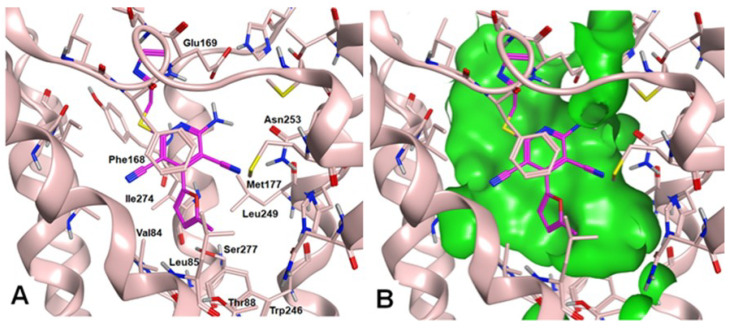
(**A,B**) Binding mode of compound **3** at the hA_2A_AR cavity. (**A**) Compound **3**–receptor interaction is shown, with indication of key receptor residues.

Compounds endowed with hA_2A_AR affinity showed a trend comparable to that observed for the hA_1_AR. Docking experiments performed at the hA_2A_AR crystal structure showed analogous arrangements of the analyzed molecules to those verified at the hA_1_AR (Figure 5 reports the binding mode of compound **3** within the hA_2A_AR cavity). The sets of residues involved in the interaction with the ligand are highly conserved at the two AR subtypes. The dicyanopyridine scaffold is inserted between Phe168, Leu249^6.51^ and Ile274^7.39^, with the exocyclic 2-amino function giving polar interaction with Glu169 and Asn253^6.55^. The 4-heterocyclic substituent is positioned close to residues belonging to TM3 (Val84^3.32^, Leu85^3.33^ and Thr88^3.36^), EL2 (Phe168), TM5 (Met177^5.38^), TM6 (Trp246^6.48^ and Leu249^6.51^) and TM7 (Ile274^7.39^, Ser277^7.42^ and His278^7.43^) domains. These residues are conserved between hA_2A_AR and hA_1_AR. Introduction of substituents on the 4-heteroaryl moiety modulates hA_2A_AR affinity to a lesser extent than what is observed for the A_1_ subtype. However, the hA_2A_AR affinity seems generally higher for compounds bearing a 4-furan-2yl moiety (compounds **1–3**) with respect to those substituted with a 4-pyridyl group. This behavior may be interpreted considering steric factors. A potential slightly smaller cavity at the hA_2A_AR, compared to that of the hA_1_AR, could accommodate slightly smaller substituents. Even the results observed for the 6-side-chain are similar to those obtained on the hA_1_AR. The hA_2A_AR residues involved in the interaction with the 6-substituent are quite conserved with the hA_1_AR subtype. In detail, the imidazolyl group gets located between residues belonging to the TM2 (Ile66^2.64^ and Ser67^2.65^), EL2 (Leu167, Phe168 and Glu169) and TM7 (Tyr271^7.36^ and Ile274^7.39^) domains. 

Generally, the introduction of substituents on the exocyclic amine at 2-position of the dicyanopyridine scaffold leads to a decrease of affinity at both AR subtypes with respect to the unsubstituted derivative **1**. Only a slight reduction of A_1_AR affinity was observed for compounds **20** and **21**. As previously observed [6], docking results on the herein reported compounds showed that substituents on the 2-amino function lead to a partial disruption of the interaction with the EL2 glutamate and TM6 asparagine residues (see above), with a consequent decrease of affinity. Furthermore, docking results suggested also various binding modes of these compounds at both the AR subtypes, making it difficult to make a clear interpretation of the biological results.

### 3.4. In Vitro Permeation Studies

The stratum corneum is a lipophilic membrane which represents the most important barrier to drug skin diffusion. In vitro permeation studies conducted on compounds **1** and **5** through Franz cells [42] showed an amount of permeated drug after 24 h respectively of 15.0 ± 1.4 μg/mL and 22.7 ± 1.0 µg/mL.

These permeation data were in agreement with the results obtained through solubility studies, carried out on both compounds in testing medium (PBS + Tween 80 (2% *w/w*)). On the basis of these experiments, compound **5** resulted in being less soluble (27.3 ± 2.52 µg/mL) than the analogous **1** (40.6 ± 0.16 µg/mL).

Therefore, it can be hypothesized that the dicyanopyridine **5**, due to its higher lipophilicity with respect to **1** (pkCSM calculated logP, 3.15 versus 2.68, respectively) [48,49] and, consequently, its affinity for stratum corneum, accumulates inside the membrane, generating an into/out concentration gradient, which represents the driving force to its permeation.

From the perspective of a possible application of the two compounds in wound healing [50] as promoters of endothelial cell proliferation and migration [21,22], the lipophilicity of compound **5** could be a desirable property, allowing an accumulation of the compound on the injured skin leading to a limited systemic absorption.

## 4. Materials and Methods

### 4.1. Chemistry

#### 4.1.1. General Methods

The microwave-assisted syntheses were performed by using an Initiator EXP Microwave Biotage instrument (frequency of irradiation: 2.45 GHz). Analytical silica gel plates (Merck F254, Kenilworth, NJ, USA), preparative silica gel plates (Merck F254, 2 mm) and silica gel 60 (Merck, 70–230 mesh) were used for analytical and preparative TLC and for column chromatography, respectively. All melting points were determined on a Gallenkamp melting-point apparatus and are uncorrected. Elemental analyses were performed with a Flash E1112 Thermofinnigan elemental analyzer for C, H and N, and the results were within ±0.4% of the theoretical values. All final compounds revealed purity not less than 95%. The IR spectra were recorded with a Perkin-Elmer Spectrum RX I spectrometer in Nujol mulls and are expressed in cm^−1^. NMR spectra were recorded on a Bruker Avance 400 spectrometer (400 MHz for ^1^H NMR and 100 MHz for ^13^C NMR). The chemical shifts are reported in δ (ppm) and are relative to the central peak of the residual non-deuterated solvent, which was CDCl_3_ or DMSO*d*_6_. The following abbreviations are used: s = singlet, d = doublet, t = triplet, q = quartet, m = multiplet, br = broad, Ar = aromatic protons. Compounds **22** [30], **25**, **26** [30], **27** [31], **29** [32], **32** [27], **35**, **36** [27], **37** [28], **39** [29], **51**, **52** and **56** were synthesized by following the procedures reported by us in Reference [6]. When available, melting point and/or ^1^H NMR values were in accordance with the literature data.

#### 4.1.2. General Procedure for the Synthesis of 2-Amino-4-(heteroaryl)-6-[(1*H*-imidazol-2-ylmethyl)sulfanyl]pyridine-3,5-dicarbonitriles **1**, **3**

Equimolar amounts of sodium hydrogen carbonate and commercially available 2-(bromomethyl)-*1H*-imidazole hydrobromide (4.0 mmol) were consequentially added to a solution of the mercapto-compound (**32** [6,27], **34**, 3.8 mmol) in anhydrous DMF (1 mL). The reaction mixture was stirred at room temperature, under a nitrogen atmosphere, for 4 h. At reaction completion, water was added (30 mL) to precipitate a solid which was collected by filtration and triturated with Et_2_O (2 mL). A suspension of the intermediate (hydrobromide salt of compounds **1** and **3**) and sodium hydrogen carbonate (4.0 mmol) in a mixture of DMF/H_2_O (2:1, 2 mL) was stirred at room temperature for a few minutes. Then the solid was collected by filtration, washed with water and recrystallized. 

2-Amino-4-(furan-2-yl)-6-[(1*H*-imidazol-2-ylmethyl)sulfanyl]pyridine-3,5-dicarbonitrile (**1**). Yield 55%; mp 233–234 °C. ^1^H NMR (DMSO-*d*_6_) 8.12 (br s, 2H, NH_2_), 8.11 (d, 1H, Ar, *J* = 1.2 Hz), 7.41 (d, 1H, Ar, *J* = 3.6 Hz), 7.11 (s, 2H, Ar), 6.84 (dd, 1H, Ar, *J* = 1.7, 3.6 Hz), 4.55 (s, 2H, CH_2_). Anal. Calc. for C_15_H_10_N_6_OS.

Hydrobromide salt of compound **1**: Yield 75%; mp > 300 °C dec (MeOH). ^1^H NMR (DMSO-*d*_6_) 13.82 (br s, 1H, NH), 8.14 (br s, 2H, NH_2_), 8.12 (d, 1H, Ar, *J* = 1.2 Hz), 7.63 (s, 2H, Ar), 7.43 (d, 1H, Ar, *J* = 3.6 Hz), 6.85 (dd, 1H, Ar, *J* = 1.7, 3.6 Hz), 4.73 (s, 2H, CH_2_); IR 3400, 3320, 3140, 2217. Anal. Calc. for C_15_H_11_BrN_6_OS.

2-Amino-6-[(1*H*-imidazol-2-ylmethyl)sulfanyl]-4-(5-methylfuran-2-yl)pyridine-3,5-dicarbonitrile (**3**): Yield 86%; mp 276–277 °C; (DMF); ^1^H NMR (DMSO-*d*_6_) 11.87 (s, 1H, NH), 8.07 (s, 2H, NH_2_), 7.35 (d, 1H, Ar, *J* = 3.5 Hz), 6.96 (s, 2H, Ar), 6.49 (d, 1H, Ar, *J* = 2.8 Hz), 4.48 (s, 2H, CH_2_), 2.40 (s, 3H, CH_3_); ^13^C NMR (DMSO-*d*_6_) 167.67, 160.83, 156.66, 143.92, 143.60, 142.99, 118.40, 116.36, 116.26, 110.00, 88.76, 81.09, 27.31, 13.88; IR 3289, 2206. Anal. Calc. for C_16_H_12_N_6_OS.

Hydrobromide salt of compound **3**: ^1^H NMR (DMSO-*d*_6_) 14.02 (br s, 1H, NH), 8.1 (br s, 2H, NH_2_), 7.60 (s, 2H, Ar), 7.38 (d, 1H, Ar, *J* = 3.4 Hz), 6.51 (d, 1H, Ar, *J* = 2.8 Hz), 4.69 (s, 2H, CH_2_), 2.40 (s, 3H, CH_3_).

#### 4.1.3. 2-Amino-4-[5-(hydroxymethyl)furan-2-yl]-6-[(1*H*-imidazol-2-ylmethyl)sulfanyl]pyridine-3,5-dicarbonitrile hydrobromide (**2**)

Sodium hydrogen carbonate (9.3 mmol) and an equimolar amount of the commercially available 2-(bromomethyl)-1*H*-imidazole hydrobromide were added to a solution of the suitable mercapto-compound (**33**, 9.3 mmol) in anhydrous DMF (3 mL). The reaction mixture was stirred at RT, in a nitrogen atmosphere, until the disappearance of the starting material (TLC monitoring). Then water was added (20 mL) to precipitate a solid which was collected by filtration and washed with water. For compound **1**, the crude product was treated with Et_2_O (2 mL), collected by filtration and recrystallized. For derivative **2**, a second crop of product was obtained by extracting the aqueous solution with EtOAc (4 × 10 mL). The collected organic layers were dried (Na_2_SO_4_), and the solvent was removed under reduced pressure. The two crops of product were triturated with Et_2_O (2 mL) collected by filtration and purified by crystallization.

Yield 55%; mp 293–294 °C; (EtOH); ^1^H NMR (DMSO-*d*_6_) 13.97 (s, 1H, NH), 8.10 (s, 2H, NH_2_), 7.58 (s, 2H, Ar), 7.38 (s, 1H, Ar), 6.66 (s, 1H, Ar), 5.49 (s, 1H, OH), 4.69 (s, 2H, CH_2_), 4.50 (s, 2H, CH_2_); ^13^C NMR (DMSO-*d*_6_) 166.10, 160.78, 160.15, 144.27, 144.02, 143.90, 119.97, 118.14, 115.96, 110.35, 89.18, 82.15, 56.22, 24.43; IR 3313, 2212, 1208. Anal. Calc. for C_16_H_13_BrN_6_O_2_S.

#### 4.1.4. General Procedure for the Synthesis of 2-Amino-4-(heteroaryl)-6-[(1H-imidazol-2-ylmethyl)sulfanyl]pyridine-3,5-dicarbonitriles **4**–**10**

Sodium hydrogen carbonate (2 mmol) and the commercially available 2-(bromomethyl)-1H-imidazole hydrobromide (1 mmol) were added to a solution of the suitable mercapto-compound (**35**–**41** [6,27,28,29] 1 mmol) in anhydrous DMF (1 mL). The reaction mixture was stirred at RT until disappearance of the starting material (TLC monitoring). Then, water was added (25 mL) to precipitate a solid which was collected by filtration and washed with water. The crude product was triturated with Et_2_O (5 mL), collected by filtration and purified by crystallization (compounds **4**–**9**) or silica gel column chromatography, eluting system cyclohexane/EtOAc/MeOH 2:6:2 (compound **10**).

2-Amino-4-(furan-3-yl)-6-[(1*H*-imidazol-2-ylmethyl)sulfanyl]pyridine-3,5-dicarbonitrile (**4**): Yield 53%; mp 271–273 °C; (EtOH); ^1^H NMR (DMSO-*d*_6_) 11.83 (s, 1H, NH), 8.26 (s, 1H, Ar), 8.02 (s, 2H, NH_2_), 7.92 (s, 1H, Ar), 7.07 (s, 1H, Ar) 6.88 (s, 1H, Ar), 6.86 (s, 1H, Ar), 4.50 (s, 2H, CH_2_); IR 3328, 2220, 2211. Anal. Calc. for C_15_H_10_N_6_OS.

2-Amino-6-[(1*H*-imidazol-2-ylmethyl)sulfanyl]-4-(thiophen-2-yl)pyridine-3,5-dicarbonitrile (**5**): Yield 76%; mp 246–248 °C; (MeOH); ^1^H NMR (DMSO-*d*_6_) 11.84 (s, 1H, NH), 8.07 (s, 2H, NH_2_), 7.96 (d, 1H, Ar, *J* = 4.28 Hz), 7.56 (d, 1H, Ar, *J* = 1.88 Hz), 7.28 (s, 1H, Ar), 6.97 (s, 2H, Ar), 4.50 (s, 2H, CH_2_); IR 3305, 2212. Anal. Calc. for C_15_H_10_N_6_S_2_.

2-Amino-6-[(1*H*-imidazol-2-ylmethyl)sulfanyl]-4-(thiophen-3-yl)pyridine-3,5-dicarbonitrile (**6**): Yield 73%; mp 239–241 °C dec; (CH_3_CN); ^1^H NMR (DMSO-*d*_6_) 11.83 (br s, 1H, NH), 8.05 (dd, 1H, Ar, *J* = 2.9, 1.2 Hz), 8.04 (br s, 2H, NH_2_), 7.78 (dd, 1H, Ar, *J* = 5.0, 2.9 Hz), 7.39 (dd, 1H, Ar, *J* = 5.0, 1.2 Hz), 7.07 (br s, 1H, Ar), 6.97 (br s, 1H, Ar), 4.50 (s, 2H, CH_2_); ^13^C NMR (DMSO-*d*_6_) 166.79, 160.36, 153.48, 142.99, 133.83, 129.32, 128.16, 127.92, 115.88, 93.29, 86.01, 27.24. IR 3400, 3321, 2218. Anal. Calc. for C_15_H_10_N_6_S_2_.

2-Amino-6-[(1*H*-imidazol-2-ylmethyl)sulfanyl]-4,4′-bipyridine-3,5-dicarbonitrile (**7**): Yield 20%; mp 230 °C dec; (EtOH); ^1^H NMR (DMSO-*d*_6_) 11.87 (br s, 1H, NH), 8.80 (d, 2H, Ar, *J* = 4.7 Hz), 8.2 (br s, 2H, NH_2_), 7.59 (d, 2H, Ar, *J* = 4.7 Hz), 7.09 (br s, 1H, Ar), 6.86 (br s, 1H, Ar), 4.52 (s, 2H, CH_2_). IR 3312, 3175, 2210. Anal. Calc. for C_16_H_11_N_7_S.

2′-Amino-6′-[(1*H*-imidazol-2-ylmethyl)sulfanyl]-3,4′-bipyridine-3′,5′-dicarbonitrile (**8**): Yield 64%; mp 244–245 °C dec; (EtOH); ^1^H NMR (DMSO-*d*_6_) 12.18 (br s, 1H, NH), 8.78–8.74 (m, 2H, Ar), 8.25 (br s, 2H, NH_2_), 8.04–8.02 (m, 1H, Ar), 7.64–7.61 (m, 1H, Ar), 7.02 (s, 2H, Ar), 4.54 (s, 2H, CH_2_). IR 3379, 3308, 2212. Anal. Calc. for C_16_H_11_N_7_S.

2′-Amino-6′-[(1*H*-imidazol-2-ylmethyl)sulfanyl]-6-methoxy-3,4′-bipyridine-3′,5′-dicarbonitrile (**9**): Yield 82%; mp 232–233 °C (EtOH); ^1^H NMR (DMSO-*d*_6_) 12.06 (s, 1H, NH), 8.38 (d, 1H, Ar, *J* = 1.7 Hz), 8.18 (s, 2H, NH_2_), 7.93 (dd, 1H, Ar, *J* = 8.6, 2.1 Hz), 7.02 (d, 1H, Ar, *J* = 8.7 Hz), 7.00 (s, 2H, Ar), 4.52 (s, 2H, CH_2_), 3.94 (s, 3H, CH_3_); ^13^C NMR (DMSO-*d*_6_) 166.66, 165.10, 160.18, 155.69, 147.37, 139.95, 123.83, 115.72, 110.98, 93.76, 86.62, 54.14, 27.21. IR 3383, 3335, 2216. Anal. Calc. for C_17_H_13_N_7_OS.

2′-Amino-6-hydroxy-6′-[(1*H*-imidazol-2-ylmethyl)sulfanyl]-3,4′-bipyridine-3′,5′-dicarbonitrile (**10**): Yield 61%; mp 269–271 °C; ^1^H NMR (DMSO-*d*_6_) 12.14 (s, 1H, OH), 11.82 (s, 1H, NH), 8.12 (s, 2H, NH_2_), 7.81 (s, 1H, Ar), 7.61 (dd, 1H, Ar, *J* = 9.5, 2.7 Hz), 7.07 (s, 1H, Ar), 6.85 (s, 1H, Ar), 6.47 (d, 1H, Ar, *J* = 9.5 Hz), 4.48 (s, 2H, CH_2_); ^13^C NMR (DMSO-*d*_6_) 166.64, 165.93, 154.78, 141.10, 115.88, 93.42, 92.66, 86.18, 83.66, 29.93, 27.15. 3400, 3308, 3185, 2214. Anal. Calc. for C_16_H_11_N_7_OS.

#### 4.1.5. 2-Amino-4-(furan-2-yl)-6-[(1*H*-imidazol-5-ylmethyl)sulfanyl]pyridine-3,5-dicarbonitrile hydrochloride (**11**)

Equimolar amounts of sodium hydrogen carbonate and the 4-(chloromethyl)-1H-imidazole hydrochloride **53** [34] (1.36 mmol) were consequentially added to a solution of the mercapto-compound **32** [6,27], (1.24 mmol) in anhydrous DMF (3 mL). The reaction mixture was stirred at RT for 3 h. At reaction completion, water was added (30 mL) to precipitate a solid which was collected by filtration, triturated with Et_2_O (2 mL) and then crystallized.

Yield 75%; mp 147–148 °C; (EtOH/Et_2_O/acetone); ^1^H NMR (DMSO-*d*_6_) 8.6–7.6 (br s, 2H, NH_2_), 8.28 (s, 1H, Ar), 8.10 (d, 1H, Ar, *J* = 1.3 Hz), 7.48 (s, 1H, Ar), 7.39 (d, 1H, Ar, *J* = 3.6 Hz), 6.84 (dd, 1H, Ar, *J* = 3.6, 1.7 Hz), 4.45 (s, 2H, CH_2_); ^13^C NMR (DMSO-*d*_6_) 167.71, 160.64, 145.52, 144.21, 116.84, 116.18, 116.13, 113.31, 89.73, 81.98, 26.02. IR 3325, 3211, 2210. Anal. Calc. for C_15_H_11_ClN_6_OS

#### 4.1.6. General Procedure for the Synthesis of 2-Amino-substituted Derivatives **12**, **13**

A solution of the phenylsulfanyl-derivative **22** [6,30] (0.47 mmol), the commercially available 1-(1*H*-imidazol-2-yl)methanamine hydrochloride for compound **12** or 2-(1*H*-imidazol-4-yl)ethanamine for **13** (0.56 mmol), and Et_3_N (1.2 mmol) in DMF (1 mL) was heated at 120 °C, under microwave irradiation, until the disappearance of the starting material (TLC monitoring). After cooling at RT, water (20 mL) was added, and the resulting precipitate was collected by filtration and washed with water (10 mL) and Et_2_O (5 mL). The crude product was purified by silica gel column chromatography, eluting system cyclohexane/EtOAc 1:1.

2-Amino-4-(furan-2-yl)-6-[(1*H*-imidazol-2-ylmethyl)amino]pyridine-3,5-dicarbonitrile (**12**): Yield 14%; mp 265–266 °C; ^1^H NMR (DMSO-*d*_6_) 11.63 (s, 1H, NH), 8.03 (s, 1H, Ar), 7.84 (t, 1H, NH, *J* = 5.5 Hz), 7.49 (s, 2H, NH_2_), 7.27 (d, 1H, Ar, *J* = 3.5 Hz), 7.04 (s, 1H, Ar), 6.82 (s, 1H, Ar), 6.79 (dd, 1H, Ar, *J* = 3.6, 1.7 Hz), 4.57 (d, 2H, CH_2_, *J* = 5.5 Hz,); ^13^C NMR (DMSO-*d*_6_) 162.02, 159.93, 145.51, 145.03, 117.29, 117.09, 115.50, 112.88, 77.55, 76.11, 65.37. IR 3337, 2203. Anal. Calc. for C_15_H_11_N_7_O.

2-Amino-4-(furan-2-yl)-6-{[2-(1*H*-imidazol-4-yl)ethyl]amino}pyridine-3,5-dicarbonitrile (**13**): Yield 37%; mp 242 °C dec;^1^H-NMR (DMSO-*d*_6_) 11.83 (br s, 1H, NH), 8.02 (d, 1H, Ar, *J* = 1.8 Hz), 7.57–7.53 (m, 2H, NH + Ar), 7.41 (br s, 2H, NH_2_), 7.25 (d, 1H, Ar, *J* = 3.6 Hz), 6.85 (s, 1H, Ar), 6.78 (dd, 1H, Ar, *J* = 3.6, 1.8 Hz), 3.62 (q, 2H, CH_2_, *J* = 7.2 Hz), 2.79 (t, 2H, CH_2_, *J* = 7.2 Hz). ^13^C NMR (DMSO-*d*_6_) 162.05; 159.92; 146.58; 146.02; 145.50; 135.08; 117.39; 117.17; 115.37; 112.81; 77.35; 75.71; 41.40. Anal. Calc. for C_16_H_13_N_7_O.

#### 4.1.7. General Procedure for the Synthesis of 2-{[6-Amino-3,5-dicyano-4-(furan-2-yl)pyridin-2-yl]sulfanyl}-*N*-acetamides **14–16**

The suitable 1-(1*H*-imidazol-2-yl)methanamine hydrocloride (compound **14**) or 1-(1*H*-imidazol-4-yl)methanamine (**55**) [36] (compound **15**) or 2-(1*H*-imidazol-4-yl)ethanamine (compounds **16**, **17**) (1.1 mmol); the intermediate compound **42** (1.0 mmol); 1-ethyl-3-(3-dimethylaminopropyl)carbodiimide hydrochloride (1.48 mmol); and 1-hydroxybenzotriazole hydrate (1.3 mmol) were subsequently added to a solution of anhydrous Et_3_N (0.5 mL) in anhydrous DMF (5 mL). The reaction mixture was stirred at RT, under a nitrogen atmosphere, until the disappearance of the starting material (TLC monitoring). After dilution with cold water (50 mL), the suspension was stirred for 15 min, and the resulting solid was collected by filtration and washed with water and Et_2_O. The crude derivative **14** was then purified by treatment with boiling EtOH (20 mL). For compound **15**, a second crop of product was obtained by extracting the aqueous phase with EtOAc (3 × 30 mL). Then the collected organic layers were anhydrified (Na_2_SO_4_) and evaporated under reduced pressure to yield a solid which was then washed with a 10% solution of Na_2_CO_3_ (30 mL) and with water. Washing with 10% solution of Na_2_CO_3_ (30 mL) was performed also on the crude compound **16**. Compounds **15** and **16** were purified by crystallization.

2-{[6-Amino-3,5-dicyano-4-(furan-2-yl)pyridin-2-yl]sulfanyl}-*N*-(1*H*-imidazol-2-ylmethyl)acetamide (**14**): Yield 72%; mp 274–276 °C; ^1^H NMR (DMSO-*d*_6_) 11.88 (br s, 1H, NH), 9.0–7.5 (br s, 2H, NH_2_), 8.60 (t, 1H, NH, *J* = 5.5 Hz), 8.11 (d, 1H, Ar, *J* = 1.3 Hz), 7.41 (d, 1H, Ar, *J* = 3.6 Hz), 6.98 (s, 2H, Ar), 6.85 (dd, 1H, Ar, *J* = 3.7, 1.7 Hz), 4.34 (d, 2H, CH_2_, *J* = 5.5 Hz), 3.91 (s, 2H, CH_2_); ^13^C NMR (DMSO-*d*_6_) 168.11, 167.43, 160.64, 145.55, 145.10, 144.09, 116.83, 116.21, 113.32, 89.40, 81.95, 37.14, 34.39. Anal. Calc. for C_17_H_13_N_7_O_2_S. 

2-{[6-Amino-3,5-dicyano-4-(furan-2-yl)pyridin-2-yl]sulfanyl}-*N*-(1*H*-imidazol-5-ylmethyl)acetamide (**15**): Yield 12%; mp 269–270 °C (EtOH). ^1^H-NMR (DMSO-*d*_6_) 11.93 (br s, 1H, NH), 8.4–7.6 (br s, 2H, NH_2_), 8.34 (t, 1H, NH), 8.12–8.11 (m, 1H, Ar), 7.61 (s, 1H, Ar), 7.41 (d, 1H, Ar, *J* = 3.6 Hz), 6.94 (s, 1H, Ar), 6.85 (dd, 1H, Ar, *J* = 3.6, 1.7 Hz), 4.21 (d, 2H, CH_2_, *J* = 5 Hz), 3.89 (s, 2H, CH_2_). Anal. Calc. for C_17_H_13_N_7_O_2_S. 

2-{[6-Amino-3,5-dicyano-4-(furan-2-yl)pyridin-2-yl]sulfanyl}-*N*-[2-(1*H*-imidazol-5-yl)ethyl]acetamide (**16**): Yield 28%; mp 223–226 °C (CH_3_CN);^1^HNMR (DMSO-*d*_6_) 11.80 (br s, 1H, NH); 8.13–8.07 (m, 2H, Ar + NH); 8.5–7.7 (s br, 2H, NH_2_), 7.50 (s, 1H, Ar); 7.41 (d, 1H, Ar, *J* = 3.6 Hz), 6.85 (dd, 1H, Ar, *J* = 3.6, 1.7 Hz), 6.82 (br s, 1H, Ar), 3.87 (s, 2H, CH_2_), 3.31–3.27 (m, 2H, CH_2_), 2.68–2.61 (m, 2H, CH_2_). ^13^C NMR (DMSO-*d*_6_) 167.74; 167.14; 160.57; 146.08; 145.54; 144.19; 116.88; 116.14; 113.33; 89.63; 82.09; 34.10. IR: 2922, 2362, 2212, 1653, 1458, 1377. Anal. Calc. for C_18_H_15_N_7_O_2_S.

#### 4.1.8. 4-({[6-Amino-3,5-dicyano-4-(furan-2-yl)pyridin-2-yl]sulfanyl}methyl)-*N*-[2-(1*H*-imidazol-5-yl)ethyl]-1,3-thiazole-2-carboxamide (**17**)

Then 1-hydroxybenzotriazole hydrate (0.9 mmol), 1-ethyl-3-(3-dimethylaminopropyl)carbodiimide hydrochloride (0.98 mmol), Et_3_N (0.85 mmol), and 2-(1*H*-imidazol-4-yl)ethanamine (2.61 mmol) were subsequently added to a solution of compound **43** (0.85 mmol) in anhydrous DMF (2 mL). The reaction mixture was stirred at RT, under a nitrogen atmosphere, for 3 h. After dilution with cold water (30 mL), a solid precipitated and was collected by filtration and washed with water and Et_2_O. A second crop of product was obtained by extracting the aqueous phase with EtOAc (3 × 30 mL). Then the collected organic layers were anhydrified (Na_2_SO_4_) and evaporated under reduced pressure to yield a solid which was treated with Et_2_O (5 mL) and then collected by filtration. The crude product was purified by silica gel column chromatography, eluting system CH_2_Cl_2_/MeOH 8:2. 

Yield 40%; ^1^H NMR (DMSO-*d*_6_) 11.85 (s, 1H, NH), 8.88 (t, 1H, NH, *J* = 5.9 Hz), 8.10 (s, 2H, Ar), 8.4–7.8 (br s, 2H, NH_2_), 7.54 (s, 1H, Ar), 7.39 (d, 1H, Ar, *J* = 3.7 Hz), 6.83 (m, 2H, Ar), 4.61 (s, 2H, CH_2_), 3.50 (dd, 2H, CH_2_, *J* = 13.7, 7.1 Hz), 2.77 (t, 2H, CH_2_, *J* = 7.3 Hz). IR 3300, 3120, 2200, 1539, 1519,1456. Anal. Calc. for C_21_H_16_N8O_2_S_2_.

#### 4.1.9. General Procedure for the Synthesis of the Target Compounds **18–21**

Sodium hydrogen carbonate (5.5 mmol) and an equimolar amount of the commercially available 2-(bromomethyl)-1*H*-imidazole hydrobromide were added to a solution of the suitable mercapto-compound (**48**–**50**, **52** [5], 5.0 mmol) in anhydrous DMF (2 mL). The reaction mixture was stirred at RT, a under nitrogen atmosphere, until the disappearance of the starting material (TLC monitoring). Then, water was added (30 mL) to precipitate a solid which was collected by filtration and washed with water. A second crop of product was obtained by extracting the aqueous solution with EtOAc (3 × 10 mL). The collected organic layers were dried (Na_2_SO_4_) and the solvent removed under reduced pressure. The two crops of product were triturated with Et_2_O (2 mL), collected by filtration and purified by crystallization.

2-(Cyclopentylamino)-4-(furan-2-yl)-6-[(1*H*-imidazol-5-ylmethyl)sulfanyl]pyridine-3,5-dicarbonitrile (**18**): Yield 73%; mp 221–222 °C (CH_3_NO_2_); ^1^H NMR (DMSO-*d*_6_) 12.62 (s, 1H, NH), 8.12 (s, 1H, Ar), 7.93 (d, 1H, NH, *J* = 7.1 Hz), 7.39 (d, 1H, Ar, *J* = 3.4 Hz), 7.09 (s, 2H, Ar), 6.85 (d, 1H, Ar, *J* = 1.8 Hz), 4.61 (s, 2H. CH_2_), 4.42 (d, 1H, CH, *J* = 6.7 Hz), 1.85 (s, 2H, CH_2_), 1.69 (s, 2H, CH_2_), 1.64–1.45 (m, 4H, 2CH_2_); ^13^C NMR (DMSO-*d*_6_): 167.39, 157.87, 147.03, 145.48, 144.14, 122.31, 116.84, 116.18, 115.96, 113.28, 89.32, 83.45, 53.68, 32.10, 27.41, 24.20; IR: 3312, 2216. Anal. Calc. for C_20_H_18_N_6_OS.

2-(Cyclopropylamino)-4-(furan-2-yl)-6-[(1*H*-imidazol-5-ylmethyl)sulfanyl]pyridine-3,5-dicarbonitrile (**19**): Yield 40%; mp 241–242 °C; (EtOH/Et_2_O); ^1^H NMR (DMSO-*d*_6_): 12.41 (s, 1H, NH), 8.36 (s, 1H, NH), 8.12 (s, 1H, Ar), 7.39 (d, 1H, Ar, *J* = 3.5 Hz), 7.04 (s, 2H, Ar), 6.85 (dd, 1H, Ar, *J* = 3.5, 1.7 Hz), 4.66 (s, 2H, CH_2_), 2.93 (s, 1H, CH), 0.77–0.70 (m, 2H, CH_2_), 0.68 (s, 2H, CH_2_); ^13^C NMR (DMSO-*d*_6_) 167.16, 159.65, 145.42, 144.09, 143.03, 122.05, 116.93, 116.10, 115.77, 113.32, 89.97, 83.55, 27.05, 25.51, 6.81; IR 3312, 2212. Anal. Calc. for C_18_H_14_N_6_OS.

4-(Furan-2-yl)-2-[(1*H*-imidazol-5-ylmethyl)sulfanyl]-6-(pyrrolidin-1-yl)pyridine-3,5-dicarbonitrile (**20**): Yield 32%; mp 232–234 °C (EtOH); ^1^H NMR (DMSO-*d*_6_) 11.94 (s, 1H, NH), 8.10 (s, 1H, Ar), 7.34 (d, 1H, Ar, J = 3.3 Hz), 7.07 (s, 1H, Ar), 6.82 (s, 2H, Ar), 4.56 (s, 2H, CH_2_), 3.82 (s, 4H, 2CH_2_), 1.96 (s, 4H, 2CH_2_); ^13^C NMR (DMSO-*d*_6_) 165.61, 156.04, 146.97, 146.33, 145.57, 122.28, 117.41, 117.03, 116.00, 113.14, 90.10, 82.88, 56.30, 50.84, 50.33, 49.63, 30.06, 27.19, 25.40; IR 2208. Anal. Calc. for C_19_H_16_N_6_OS.

*N*-{3,5-dicyano-4-(furan-2-yl)-6-[(1*H*-imidazol-2-ylmethyl)sulfanyl]pyridin-2-yl}acetamide (**21**): Yield 27%; mp 203–204 °C (EtOH/Et_2_O); ^1^H NMR (DMSO-*d*_6_) 12.28 (s, 1H, NH), 11.20 (s, 1H, NH), 8.21 (d, 1H, Ar, *J* = 4.2 Hz), 7.55 (d, 1H, Ar, *J* = 3.9 Hz), 7.04 (s, 2H, Ar), 6.96–6.77 (m, 1H, Ar), 4.65 (d, 2H, CH_2_, *J* = 4.2 Hz), 2.24 (d, 3H, CH_3_, *J* = 4.8 Hz,); ^13^C NMR (DMSO-*d*_6_) 169.70, 167.18, 155.21, 148.16, 144.88, 144.44, 142.59, 122.63, 118.47, 115.13, 114.88, 113.86, 98.26, 94.65, 27.32, 24.24. IR 3219, 2231, 2214, 1710, 1604. Anal. Calc. for C_17_H_12_N_6_O_2_S.

#### 4.1.10. General Procedure for the Synthesis of 2-Amino-4-heteroaryl-6-(phenylsulfanyl)pyridine-3,5-dicarbonitriles **23**, **24**, **30**

A solution of the suitable aldehyde (10 mmol), malononitrile (20 mmol) and tetrabutylammonium fluoride hydrate (10% mol) in water (50 mL) was stirred at RT for 20 min. Then thiophenol (10 mmol) was added and the mixture was heated at 80 °C for 2 h. After cooling at RT, the water was removed under reduced pressure, the residue dissolved in EtOAc (100 mL) and the resulting solution dried (Na_2_SO_4_). After distillation of the solvent, the crude product was triturated with a mixture of Et_2_O/EtOH (10:1), collected by filtration and recrystallized. 

2-Amino-4-[5-(hydroxymethyl)furan-2-yl]-6-(phenylsulfanyl)pyridine-3,5-dicarbonitrile (**23**): Yield 22%; mp 161–162 °C (CH_3_NO_2_). ^1^H NMR (DMSO-*d*_6_) 7.78 (br s, 2H, NH_2_), 7.60 (dd, 2H, Ar, *J* = 7.0, 2.4 Hz), 7.53–7.44 (m, 3H Ar), 7.37 (d, 1H, Ar, *J* = 3.5 Hz), 6.66 (d, 1H, Ar, *J* = 3.5 Hz); 5.51 (t, 1H, OH, *J* = 5.8 Hz), 4.52 (d, 2H, CH_2_, *J* = 5.8 Hz). IR 3414, 3296, 2206, 1026. Anal. Calc. for C_18_H_12_N_4_O_2_S.

2-Amino-4-(5-methylfuran-2-yl)-6-(phenylsulfanyl)pyridine-3,5-dicarbonitrile (**24**): Yield 14%; mp 170–172 °C (EtOH); ^1^H NMR (DMSO *d*_6_) 7.72 (br s, 2H, NH_2_); 7.63–7.55 (m, 2H, Ar), 7.49 (d, 3H, Ar, *J* = 49 Hz), 7.36 (d, 1H, Ar, *J* = 3.3 Hz), 6.50 (d, 1H Ar, *J* = 2.7 Hz), 2.41 (s, 3H CH_3_), IR 3456, 3331, 2214. Anal. Calc. for C_18_H_12_N_4_OS.

2′-Amino-6-methoxy-6′-(phenylsulfanyl)-3,4′-bipyridine-3′,5′-dicarbonitrile (**30**): Yield 22%; mp 244–245 °C (CH_3_NO_2_); ^1^H NMR (DMSO-*d*_6_) 8.41 (d, 1H, Py, *J* = 2.3 Hz), 7.96 (dd, 1H, Py, *J* = 8.6, 2.5 Hz), 7.87 (br s, 2H, NH_2_), 7.61 (dd, 2H, Ar, *J* = 6.4, 2.9Hz), 7.53–7.47 (m, 3H, Ar), 7.06 (d, 1H, Py, *J* = 8.6 Hz), 3.95 (s, 3H, CH_3_). IR 3448, 3344, 3230, 2214. Anal. Calc. for C_19_H_13_N_5_OS.

#### 4.1.11. The 2-Amino-6-(phenylsulfanyl)-4,4′-bipyridine-3,5-dicarbonitrile (**28**)

A suspension of the suitable aldehyde (8.9 mmol), malononitrile (20.8 mmol), thiophenol (10.4 mmol) and basic alumina (0.019 mol) in water (20 mL) was stirred at 100 °C until the disappearance of the starting material (TLC monitoring). After cooling at RT, the aqueous layer was separated by decantation from the yellow sticky mass. The residue was treated with a mixture EtOH/Et_2_O 1:2 (3 mL), yielding a solid which was then collected by filtration and extracted with boiling EtOAc (100 mL×2). After distillation of the combined and dried (Na_2_SO_4_) organic layers, the crude product was triturated with a little of 1:1 mixture of Et_2_O/EtOH and collected by filtration. The compound was used without further purification. Yield 70%; mp > 300 °C. ^1^H NMR (DMSO-*d*_6_) 8.82 (d, 2H, Ar, *J* = 4.7 Hz), 7.95 (br s, 2H, NH_2_), 7.64–7.58 (m, 4H, Ar), 7.55–7.49 (m, 3H, Ar) [31]. IR 3335, 3224, 2223. 

#### 4.1.12. The 2′-Amino-6-hydroxy-6′-(phenylsulfanyl)-3,4′-bipyridine-3′,5′-dicarbonitrile (**31**)

A solution 33% HBr in acetic acid (8 mL) containing **30** (1.7 mmol) was heated at 70 °C for 8 h. After evaporation of the solvent under reduced pressure, a yellow solid was obtained and was then treated with Et_2_O (10 mL) and collected by filtration. 

Yield 76%; mp > 300 °C (CH_3_NO_2_). ^1^H NMR (DMSO-*d*_6_) 7.86–7.85 (m, 1H, Ar), 7.80 (br s, 2H, NH_2_), 7.64 (dd, 1H, Ar, *J* = 9.5, 2.2 Hz), 7.60–7.54 (m, 2H, Ar), 7.50 (d, 3H, Ar, *J* = 4.9 Hz), 6.49 (d, 1H, Ar, *J* = 9.5 Hz); IR 3323, 3221, 2214. Anal. Calc. for C_18_H_11_N_5_OS.

#### 4.1.13. General Procedure for the Synthesis of 2-Amino-4-(heteroaryl)-6-sulfanylpyridine-3,5-dicarbonitriles **33**, **34**, **38**, **40**, **41**

To a stirred solution of the suitable phenyl-sulfanyl derivative **23**, **24, 28** [6,31], **30** and **31** (10 mmol) in anhydrous DMF (1 mL) maintained at RT and under nitrogen atmosphere, an excess of anhydrous sodium sulfide (33 mmol) was added. The reaction mixture was heated at 80 °C for 2 h. Then 1N HCl (25 mL), was drop-by-drop added to obtain a precipitate which was then collected by filtration and washed with water (20 mL) and Et_2_O (5 mL). Compounds **33**, **34**, **38** and **41** were recrystallized, while derivative **40** was purified by silica gel column chromatography, eluting system: cyclohexane/MeOH/EtOAc, 2:2:6. 

2-Amino-4-[5-(hydroxymethyl)furan-2-yl]-6-sulfanylpyridine-3,5-dicarbonitrile (**33**): Yield 97%; mp 280–282 °C (EtOH/2-Methoxyethanol). ^1^H NMR (DMSO-*d*_6_) 12.90 (br s, 1H, SH), 7.85 (br s, 2H, NH_2_), 7.40 (d, 1H, Ar, *J* = 3.5 Hz), 6.65 (d, 1H, Ar, *J* = 3.4 Hz), 5.50 (br s, 1H, OH), 4.51 (s, 2H, CH_2_). IR 3286, 3213, 2204. Anal. Calc. for C_12_H_8_N_4_O_2_S.

2-Amino-4-(5-methylfuran-2-yl)-6-sulfanylpyridine-3,5-dicarbonitrile (**34**): Yield 96%; mp > 300 °C; (EtOH/2-Methoxyethanol). ^1^H NMR (DMSO-*d*_6_) 12.85 (br s, 1H, SH), 7.81 (br s, 2H, NH_2_), 7.41 (d, 1H, Ar, *J* = 3.2 Hz), 6.51 (d, 1H, Ar, *J* = 2.9 Hz), 2.41 (s, 3H, CH_3_); IR 3383, 3298, 2214. Anal. Calc. for C_12_H_8_N_4_OS.

2-Amino-6-sulfanyl-4,4′-bipyridine-3,5-dicarbonitrile (**38**): Yield 89%; mp > 300 °C (MeOH); ^1^HNMR (DMSO-*d*_6_) 13.2 (br s, 1H, SH); 8.77 (d, 2H, Ar, *J* = 4.7 Hz); 7.53 (d, 2H, Ar, *J* = 4.7 Hz); IR 3310, 3193, 2225. Anal. Calc. for C_12_H_7_N_5_S.

2′-Amino-6-methoxy-6′-sulfanyl-3,4′-bipyridine-3′,5′-dicarbonitrile (**40**): Yield 50%; mp > 300 °C; ^1^H NMR (DMSO-*d*_6_) 13.06 (br s, 1H, SH), 8.34 (s, 1H, Ar), 7.88 (d, 1H, Ar, *J* = 8.6 Hz), 7.71 (br s, 2H, NH_2_), 7.00 (d, 1H, Ar, *J* = 8.6 Hz), 3.94 (s, 3H, CH_3_); IR 3315, 3209, 2216. Anal. Calc. for C_13_H_9_N_5_OS.

2′-Amino-6-hydroxy-6′-sulfanyl-3,4′-bipyridine-3′,5′-dicarbonitrile (**41**): Yield 74%; mp > 300 °C (MeOH). ^1^H NMR (DMSO-*d*_6_) 12.98 (br s, 1H, SH), 12.16 (br s, 1H, OH), 7.87 (br s, 2H, NH_2_), 7.81 (s, 1H, Ar), 7.59 (dd, 1H, Ar, *J* = 9.5, 2.5 Hz), 6.47 (d, 1H, Ar, *J* = 9.5 Hz). IR 3288, 3172, 2208. Anal. Calc. for C_12_H_7_N_5_OS.

#### 4.1.14. {[6-Amino-3,5-dicyano-4-(furan-2-yl)pyridin-2-yl]sulfanyl}acetic acid (**42**)

A suspension of **32** (1.44 mmol) in anhydrous DMF (2 mL) containing NaHCO_3_ (2.9 mmol) was stirred at RT, under a nitrogen atmosphere, for 30 min. After an addition of chloroacetic acid (1.44 mmol), the reaction mixture was stirred at RT, under a nitrogen atmosphere, for 5 h. The resulting brown suspension was diluted with water (30 mL) and acidified (pH 2) with 6N HCl. A solid precipitate was collected by filtration and washed with water. The product was used for the next step, without further purification. 

Yield 80%; mp 260–262 °C; ^1^H-NMR (DMSO-*d*_6_) 12.97 (br s, 1H, OH), 8.12 (d, 1H, Ar, *J* = 1.2 Hz), 8.00 (br s, 2H, NH_2_), 7.41 (d, 1H, Ar, *J* = 3.6 Hz), 6.85 (dd, 1H, Ar, *J* = 1.7, 3.6 Hz), 4.12 (s, 2H, CH_2_). 

#### 4.1.15. 4-({[6-Amino-3,5-dicyano-4-(furan-2-yl)pyridin-2-yl]sulfanyl}methyl)-1,3-thiazole-2-carboxylic acid (**43**)

Sodium hydrogen carbonate (7.5 mmol) and an equimolar amount of compound **57** [35] were added to a solution of the mercapto-derivative **32** (6.8 mmol) in anhydrous DMF (2 mL). The reaction mixture was stirred at RT under nitrogen atmosphere until the disappearance of the starting material (TLC monitoring). Then water was added (30 mL) to precipitate a solid which was collected by filtration and washed with water. A second crop of product was obtained by extracting the aqueous solution with EtOAc (3 × 30 mL). The collected organic layers were washed with brine (3 × 30 mL), dried (Na_2_SO_4_) and the solvent was removed under reduced pressure. The crude product was used for the next step, without further purification.

Yield 30%; ^1^H NMR (DMSO-*d*_6_) 8,16 (s, 1H, Ar), 8.10 (d, 1H, Ar, *J* = 1.3 Hz), 7.40 (d, 1H, Ar, *J* = 3.7 Hz), 6.84 (dd, 1H, Ar, *J* = 3.6, 1.8 Hz), 4.64 (s, 2H, CH_2_). 

#### 4.1.16. 2-Chloro-4-(furan-2-yl)-6-(phenylsulfanyl)pyridine-3,5-dicarbonitrile (**44**)

A suspension of CuCl_2_ (56.5 mmol) and isoamyl nitrite (56.5 mmol) in anhydrous acetonitrile (10 mL) was stirred at RT for 20 min. After the addition of the 2-amino-derivative **22** [30] (94.2 mmol), the mixture was then left under stirring at RT and nitrogen atmosphere for 48 h. Then it was acidified with 1N HCl and extracted with EtOAc (50 mL × 3). The collected organic layers were washed with water (100 mL × 2) to a neutral pH and dried over Na_2_SO_4_. The solvent was evaporated under reduced pressure, yielding a solid which was recovered with Et_2_O (5 mL) and collected by filtration. 

Yield 57%; mp 196–198 °C (EtOH); ^1^H NMR (DMSO-*d*_6_) 8.30 (d, 1H, Ar, *J* = 1.7 Hz), 7.70 (d, 1H, Ar, *J* = 3.7 Hz), 7.64 (dd, 2H, Ar, *J* = 6.4, 1.5 Hz), 7.58 (t, 3H, Ar, *J* = 6.3 Hz), 6.97 (dd, 1H, Ar, *J* = 3.7, 1.7 Hz); IR 2232. Anal. Calc. for C_17_H_8_ClN_3_OS.

#### 4.1.17. General Procedure for the Synthesis of 2(6)-Substituted-4-(furan-2-yl)-6(2)-(phenylsulfanyl)pyridine-3,5-dicarbonitriles **45–47**

A solution of the 2-chloro-derivative **44** (7.4 mmol) and the suitable amine (14.8 mmol) in anhydrous DMF (2 mL) was left under stirring at RT and a nitrogen atmosphere for 2 h. Then water (50 mL) was added, and the mixture was stirred at RT for 10 min. The resulting precipitate was collected by filtration, washed with water and then with a mixture of diethyl ether and petroleum ether.

2-(Cyclopentylamino)-4-(furan-2-yl)-6-(phenylsulfanyl)pyridine-3,5-dicarbonitrile (**45**): Yield 79%; mp 224–225 °C; (EtOH); ^1^H NMR (CDCl_3_): 7.74 (d, 1H, Ar, *J* = 1.7 Hz), 7.61–7.58 (m, 3H, Ar), 7.48–7.44 (m, 3H, Ar), 6.66 (dd, 1H, Ar, *J* = 3.6, 1.7 Hz), 5.59 (d, 1H, NH, *J* = 6.3 Hz), 3.70–3.63 (m, 1H, CH), 1.65–1.60 (m, 4H, CH), 1.45–1.41 (m, 2H, CH), 1.23–1.20 (m, 2H, CH); IR 3317, 2208. Anal. Calc. for C_22_H_18_N_4_OS.

2-(Cyclopropylamino)-4-(furan-2-yl)-6-(phenylsulfanyl)pyridine-3,5-dicarbonitrile (**46**): Yield 94%; mp 199–200 °C (EtOH); ^1^H NMR (CDCl_3_): 7.74 (s, 1H, Ar), 7.63–7.61 (m, 2H, Ar), 7.57 (d, 1H, Ar, *J* = 3.6 Hz), 7.47–7.43 (m, 3H, Ar), 6.67 (dd, 1H, Ar, *J* = 3.6, 1.7 Hz), 5.8 (br s, 1H, NH) 2.34–2.30 (m, 1H, CH), 0.45– 0.42 (m, 2H, CH), 0.39–0.37 (m, 2H, CH); IR 3327, 2210. Anal. Calc. for C_20_H_14_N_4_OS.

4-(Furan-2-yl)-2-(phenylsulfanyl)-6-(pyrrolidin-1-yl)pyridine-3,5-dicarbonitrile (**47**): Yield 84%; mp 172–173 °C (EtOH); ^1^H NMR (CDCl_3_): 7.73 (d, 1H, Ar, *J* = 1.7 Hz), 7.59–7.57 (m, 2H, Ar), 7.48–7.43 (m, 3H, Ar), 7.39 (d, 1H, Ar, *J* = 3.6 Hz), 6.65 (dd, 1H, Ar, *J* = 3.6, 1.7 Hz), 3.6–3.3 (br s, 4H, CH), 1.9–1.8 (br s, 4H, CH); IR 2210. Anal. Calc. for C_21_H_16_N_4_OS.

#### 4.1.18. General Procedure for the Synthesis of 2(6)-Substituted-4-(furan-2-yl)-6(2)-sulfanylpyridine-3,5-dicarbonitriles **48–50**

An excess of sodium sulfide (5.6 mmol) was added to a solution of the suitable phenylsulfanyl derivative **45**–**47** (1.9 mmol) in anhydrous DMF (2 mL). The reaction mixture was stirred at 80 °C, under a nitrogen atmosphere, for 2 h. Then 1 N HCl (30 mL), followed by 6 N HCl (1 mL), was dropwise added to the cold solution to yield a solid which was then collected by filtration and washed with water (50 mL) and Et_2_O (5 mL). The residue was suspended in a mixture of petroleum ether and Et_2_O (30 mL, 1:1) and maintained under stirring for 30 min. Thus, the orange solid was collected by filtration and washed with a little of petroleum ether. 

2-(Cyclopentylamino)-4-(furan-2-yl)-6-sulfanylpyridine-3,5-dicarbonitrile (**48**): Yield 95%; mp 219–220 °C (MeOH); ^1^H NMR (DMSO-*d*_6_) 8.10 (s, 1H, Ar), 8.06 (br s, 1H, NH), 7.34 (d, 1H, Ar, *J* = 3.5 Hz), 6.82 (s, 1H, Ar), 4.54–4.51 (m, 1H, CH), 2.00–1.93 (m, 2H, CH), 1.74–1.55 (m, 6H, CH); IR 3312, 2212. Anal. Calc. for C_16_H_14_N_4_OS. 

2-(Cyclopropylamino)-4-(furan-2-yl)-6-sulfanylpyridine-3,5-dicarbonitrile (**49**): Yield 89%; mp 265–266 °C; (EtOH/2-Methoxyethanol); ^1^H NMR DMSO-*d*_6_) 8.84 (s, 1H, NH), 8.11 (s, 1H, Ar), 7.36 (d, 1H, Ar, *J* = 3.5 Hz), 6.83 (dd, 1H, Ar, *J* = 3.5, 1.6 Hz), 2.97 (d, 1H, CH, *J* = 2.2 Hz), 0.95–0.86 (m, 2H, CH), 0.79 (d, 2H, CH, *J* = 2.8 Hz); IR 3231, 2220. Anal. Calc. for C_14_H_10_N_4_OS.

4-(Furan-2-yl)-2-(pyrrolidin-1-yl)-6-sulfanylpyridine-3,5-dicarbonitrile (**50**): Yield 78%; mp 175–176 °C; (EtOH); ^1^H NMR (DMSO-*d*_6_) 8.07 (s, 1H, Ar), 7.29 (d, 1H, Ar, *J* = 3.3 Hz), 6.80 (dd, 1H, Ar, *J* = 3.4, 1.6 Hz), 3.82 (br s, 4H, CH), 1.95 (br s, 4H, CH); IR 2220. Anal. Calc. for C_15_H_12_N_4_OS. 

#### 4.1.19. The 4-(Chloromethyl)-1*H*-imidazole Hydrochloride (**53**) 

A solution of commercially available 1*H*-imidazol-4-ylmethanol (8.15 mmol) in a large excess of thionyl chloride (8 mL) was heated at reflux for 3 h. After evaporation under reduced pressure of the excess of the reagent, cyclohexane (10 mL ×2) was added to the residue and then removed under reduced pressure to yield the crude product. The latter was then treated with diethyl ether (5 mL), collected by filtration and used without further purification.

Yield 93%; mp 141–143 °C; ^1^H NMR (DMSO-*d*_6_) 9.15 (s, 1H, Ar), 7.75 (s, 1H, Ar), 4.89 (s, 2H, CH_2_) [34]. 

#### 4.1.20. The 4-(Azidomethyl)-1*H*-imidazole (**54**) 

A suspension of 4-(chloromethyl)-1*H*-imidazole (**53**) [34] (7.2 mmol) and sodium azide (21.6 mmol) in ethanol (10 mL) and DMF (0.1 mL) was heated at 70 °C, under stirring, for 12 h. After cooling at room temperature and removing by filtration of the resulting solid, the filtrate was evaporated under reduced pressure to yield an oily residue which was then purified by silica gel column chromatography, eluting system CH_2_Cl_2_/MeOH 9:1. Yield 30%; ^1^H-NMR (DMSO-*d*_6_) 12.11 (br s, 1H, NH), 7.66 (s, 1H, Ar), 7.15 (s, 1H, Ar), 4.27 (s, 2H, CH_2_) [36].

#### 4.1.21. The 1-(1*H*-Imidazol-4-yl)methanamine (**55**) 

A solution of the azido derivative **54** in ethanol containing Pd/C (10%, 150 mg) was hydrogenated in the Parr apparatus at 15 Psi for 12 h. After removing of the catalyst by filtration, the solvent was distilled under reduced pressure to yield a solid which was utilized for the next step, without further purification. Yield 88%; ^1^H-NMR (CDCl_3_) 7.60 (s, 1H, Ar), 6.91 (s, 1H, Ar), 3.89 (s, 2H, CH_2_) [36]. 

#### 4.1.22. Ethyl 4-(Chloromethyl)-1,3-thiazole-2-carboxylate (**56**) 

A solution of ethyl 2-amino-2-thioxoacetate (11.3 mmol) and 1,3-dichloroacetone (14.7 mmol) in anhydrous acetone (20 mL) was heated at reflux under stirring and nitrogen atmosphere for 24 h. The resulting solution was concentrated to a small volume and then diluted with EtOAc (30 mL). The mixture was washed with a saturated solution of NaHCO_3_ (30 mL × 3) and water (30 mL × 3), dried (Na_2_SO_4_) and evaporated under reduced pressure to afford an orange oil. Yield 87%; ^1^H NMR (DMSO-*d*_6_): 8.17 (s, 1H, Ar), 4.92 (s, 2H, CH_2_), 4.39 (q, 2H, CH_2_, *J* = 7.1 Hz), 1.34 (t, 3H, CH_3_, *J* = 7.1 Hz). IR 1732, 1712 [35]. 

#### 4.1.23. The 4-(Chloromethyl)-1,3-thiazole-2-carboxylic acid (**57**) 

An excess of lithium hydroxide monohydrate (18.8 mmol) was added to a solution of the ethyl carboxylate ester (**56**) [35] (8.42 mmol) in a mixture of acetonitrile and water (150 mL, 1:1). The reaction mixture was stirred at RT until the disappearance of the starting material (10–60 min), and then, after ice-cooling, a 1M solution of citric acid was dropwise added up to pH = 2–3. After stirring for 1 h, the resulting solution was extracted with EtOAc (140 mL × 3), and the collected organic layers were washed with water (420 mL × 3) up to the neutrality, dried (Na_2_SO_4_) and evaporated under reduced pressure. The resulting oily residue was used without further purification. Yield 44%; ^1^H NMR (DMSO-*d*_6_) 14.14 (br s, 1H, OH), 8.11 (s, 1H, Ar), 4.9 (s, 2H, CH_2_) [35]. 

### 4.2. Pharmacological Assays

#### 4.2.1. Cell Culture and Membrane Preparation

CHO cells transfected with hA_1_, hA_2A_, hA_2B_ and hA_3_ ARs (Perkin Elmer, Boston, MA, USA) were grown adherently and maintained in Dulbecco’s modified Eagle’s medium with nutrient mixture F12, containing 10% fetal calf serum, penicillin (100 U/mL), streptomycin (100 μg/mL), l-glutamine (2 mM) and geneticine (G418; 0.2 mg/mL), at 37 °C, in 5% CO_2_/95% air, until their use in cAMP assays [51]. For membrane preparation, the culture medium was removed, and the cells were washed with phosphate-buffered saline and scraped off T75 flasks in ice-cold hypotonic buffer (5 mM Tris HCl, 1 mM EDTA, pH 7.4). The cell suspension was homogenized with a Polytron, centrifuged for 30 min at 40,000× *g*, at 4 °C, and the resulting membrane pellet was used for competition binding experiments [51].

#### 4.2.2. Competition Binding Experiments

All synthesized compounds were tested for their affinity to hA_1_, hA_2A_ and hA_3_ ARs. Competition experiments to hA_1_AR were carried out by incubating 1 nM [^3^H]-8-cyclopentyl-1,3-dipropylxanthine ([^3^H]-DPCPX) with membrane suspension (50 μg of protein/100 μL) and different concentrations of the examined compounds at 25 °C for 90 min in 50 mM Tris HCl, pH 7.4. Non-specific binding was defined as binding in the presence of 1 μM DPCPX and was always <10% of the total binding [51]. Inhibition experiments to hA_2A_ ARs were performed by incubating the radioligand [^3^H]-4-(-2-[7-amino-2-{2-furyl}{1,2,4}triazolo{2,3-a} {1,3,5}triazin-5-yl-amino]ethyl)phenol ([^3^H]-ZM241385) (1 nM) with the membrane suspension (50 μg of protein/100 μL) and different concentrations of the examined compounds for 60 min, at 4 °C, in 50 mM Tris HCl (pH 7.4), 10 mM MgCl_2_. Non-specific binding was determined in the presence of ZM241385 (1 μM) and was about 20% of the total binding [52]. Competition binding experiments to A_3_ARs were carried out by incubating the membrane suspension (50 μg of protein/100 μL) with 0.5 nM [^125^I]-*N*6-(4-aminobenzyl)-*N*-methylcarboxamidoadenosine ([^125^I]-ABMECA) in the presence of different concentrations of the examined compounds for an incubation time of 120 min, at 4 °C, in 50 mM Tris HCl (pH 7.4), 10 mM MgCl_2_, 1 mM EDTA. Non-specific binding was defined as binding in the presence of 1 μM ABMECA and was always <10% of the total binding [53]. Bound and free radioactivity were separated by filtering the assay mixture through Whatman GF/B glass-fiber filters, using a Brandel cell harvester (Brandel Instruments, Unterföhring, Germany). The filter bound radioactivity was counted by Packard Tri Carb 2810 TR scintillation counter (Perkin Elmer).

#### 4.2.3. Cyclic AMP Assays

CHO cells transfected with hAR subtypes were washed with phosphate-buffered saline, detached with tripsine and centrifuged for 10 min at 200× *g*. Cells were seeded in a 96-well white half-area microplate (Perkin Elmer, Boston, USA) in a stimulation buffer composed of Hank Balanced Salt Solution, 5 mM HEPES, 0.5 mM Ro 20–1724, 0.1% BSA. The cAMP levels were then quantified by using the AlphaScreen cAMP Detection Kit (Perkin Elmer, Boston, MA, USA), following the manufacturer’s instructions [54]. At the end of the experiments, the plates were read with a Perkin Elmer EnSight Multimode Plate Reader.

#### 4.2.4. Data Analysis

The protein concentration was determined according to a Bio-Rad method, with bovine albumin as a standard reference. Inhibitory binding constant (*K_i_*) values were calculated from those of IC_50_ according to the Cheng–Prusoff equation, *K_i_* = IC_50_/(1 + [C*]/KD*), where [C*] is the concentration of the radioligand, and KD* its dissociation constant [53]. *K_i_* and IC_50_ values were calculated by non-linear regression analysis, using the equation for a sigmoid concentration-response curve (Graph-PAD Prism, San Diego, CA, USA).

### 4.3. Ab Initio Quantum Mechanical Studies

The ab initio quantum mechanical calculations were performed by using the program GAMESS (General Atomic and Molecular Electronic Structure System) [37], a general ab initio quantum chemistry package which is maintained by the members of the Gordon research group (Department. of Chemistry) at Iowa State University, USA, which uses an RHF (Restricted Hartree-Fock) calculation and whose basic function for the description of atomic orbitals is average accuracy (basis set 3–21 G) [37,55,56]

### 4.4. Molecular Modeling

Receptor refinement and energy minimization tasks were carried out by using Molecular Operating Environment (MOE, version 2019.01) suite [41]. Docking experiments were performed with CCDC Gold [40]. 

A_1_AR and A_2A_AR crystal structures refinement: The recently published cryo-EM structure of the h_A1_AR and the crystal structure of the human A_2A_AR in complex with adenosine and ZM241385, respectively, were downloaded by the Protein Data Bank webpage (http://www.rcsb.org; accessed on 6 April 2012, pdb code, 6D9H, with 3.6-Å resolution [38]; and pdb code, 4EIY, with 1.8-Å resolution [39], respectively). Both structures were checked within MOE and corrected by restoring missing loops and the wild-type receptor sequences and by adding hydrogen atoms. The Homology Modeling tool of MOE was used for these tasks. The protein structures were then energetically minimized with MOE, using the AMBER99 force field, until the RMS gradient of the potential energy was less than 0.05 kJ mol-1 Å-1. The reliability and quality of the models were checked by using the Protein Geometry Monitor application within MOE. 

Molecular docking analysis: Docking analyses were performed by using CCDC Gold [40], with default efficiency settings through MOE interface, by selecting ChemScore as scoring function and 50 poses to be generated for each ligand. Each docking pose was then energetically minimized within the respective receptor target within MOE by keeping fixed the receptor coordinates. The minimized docking poses were then re-scored by using ChemScore as the scoring function.

### 4.5. Permeation Studies

#### 4.5.1. HPLC Assay

Compound assay was performed by HPLC (Merck Hitachi Elite LaChrom apparatus, Darmstadt, Germany), equipped with a L-2400 UV–Vis detector and an L-2130 isocratic pump. The mobile phase A was water, and the mobile phase B was methanol. Gradient steps were programmed as follows: A–B 60%–40% at time 0, ramped to 70% B in 3 min, held at 70% for 2 min and returned to initial conditions during 3 min. A Hibar Purospher^®^ RP-8e (150 × 4.6 mm, 5 µm pore size) was the stationary phase. UV detection was performed at 292 nm. The injection volume was 20 µL, the flow rate was 1.2 mL/min and the column temperature was 40 ± 1 °C. Under these conditions, the compounds retention time was 5.30 ± 0.01 min. The method was validated for linearity (r^2^ = 0.998; r^2^ = 0.997), limit of quantification (3.03 µg/mL; 4.50 µg/mL) and limit of detection (0.91 µg/mL; 1.35 µg/mL) for P293BL and P297, respectively. 

#### 4.5.2. Evaluation of In Vitro Permeation

In vitro permeation studies were carried out by using vertical Franz diffusion cell [42] (Rofarma, Gaggiano, Italy). Artificial membranes of cellulose nitrate with a pore size of 0.45 µm (Sartorius, Gӧttingen, Germany) impregnated with lauryl alcohol as lipid phase simulating the epidermal barrier were employed for the study. Briefly, each membrane was weighted, completely saturated with lauryl alcohol, dried by a filter paper, then weighted again to check the weight increase and immediately mounted on cell. The acceptor medium, maintained at 37 °C and kept under gentle agitation with a magnetic bar at 50 rpm, consisted of pH 7.4 phosphate buffer (PBS) containing Tween 80 (2% *w/w*) to increase the compounds’ solubility in order to maintain sink conditions. A fixed amount (2 mL) of compounds solution (PBS pH 7.4 and Tween 80 2% *w/w*) was placed in the donor compartment. At predetermined time intervals (1, 2, 3, 4, 5, 6 and 24 h), 0.5 mL samples were withdrawn from the receiving chamber, and the drug concentration was assayed by HPLC. A correction for the cumulative dilution, due to the sample replacement with an equal volume of fresh medium, was calculated. All the experiments were performed in triplicate.

## 5. Conclusions

This study has produced a new set of amino-3,5-dicyanopyridines as AR ligands which were synthesized to deepen the SARs of this versatile series. With this in mind, molecular modeling studies supported the rationalization of the biological results obtained on this set. In general, the target compounds interacted better with both the hA1 and A2A subtypes than with the other ARs. However, compounds **1** and **5** emerged as pan ligands by binding all the subtypes with similar binding affinity in the nanomolar range. This interesting behavior, together with their partial agonist profile, suggested the need to evaluate them for their potential use in wound healing. Preliminary results on their permeation capability through artificial membrane, simulating the epidermal barrier, indicated that compound **5** could be a candidate for further evaluation as a promoter of skin-wound healing.

## Figures and Tables

**Table 3:** Formation energies of selected dicyanopyridine derivatives and evaluation of the relative intramolecular H-bond energy.

Compd	Formation Energies		
	Conformation 1 (Intramolecular H-Bond)	Conformation 2 (No Intramolecular H-Bond	Intramolecular H-Bond
	E_1_ (Hartree)	E_2_ (Hartree)	ΔE = E_1_ − E_2_ (Kcal/mol)
**4**	−1478.7221	−1478.7188	−2.1
**6**	−1686.585	−1686.585	0.0
**7**	−1383.427	−1383.4216	−3.4
**10**	−1457.8725	−1457.8675	−3.1
**12**	−1024.6594	−1024.6409	−11.6

## Data Availability

Data is contained within the article and supplementary material.

## Supplementary Materials

The following are available online at https://www.mdpi.com/article/10.3390/ph15040478/s1. Figures S1–S6 report on the different ^1^H NMR spectra registered in DMSO-*d*_6_ for compound **10** [57,58,59]. Figure S1: Before purification (crude product). Figure S2: After purification (crystallization). Figure S3: Immediately after adding a drop of D_2_O (crystallized product). Figure S4: Twenty-four hours after adding a drop of D_2_O (crystallized product). Figure S5: Forty-eight hours after adding a drop of D_2_O (crystallized product). Figure S6: One week after adding a drop of D_2_O (crystallized product). Table S1: Combustion analysis data of the newly synthesized compounds **1**–**21**, **23_,_ 24**, **30_,_ 31**, **33**, **34**, **38**, **40**, **41** and **44**–**50**.

## Abbreviations

AB-MECA: *N*6-(4-Aminobenzyl)-*N*-methylcarboxamidoadenosine; AC, adenylate cyclase; AR, adenosine receptor; BSA, bovine serum albumin; CCPA, 2-chloro-*N*6-cyclopentyladenosine; CHO, Chinese Hamster Ovary; DPCPX, 8-cyclopentyl-1,3-dipropylxanthine; EL, extracellular loop; EtOAc, ethyl acetate; DMF, dimethylformamide; DMSO, dimethylsulfoxide; EDTA, ethylendiaminotetracetic acid; GAMESS, General Atomic and Molecular Electronic Structure System; HEPES, 4-(2-hydroxyethyl)-1-piperazine-1-ethane sulfonic acid; MOE, molecular operating environment; RT, room temperature; RHF, Restricted Hartree-Fock; SARs, structure-activity relationships; THF, tetrahydrofuran; TM, transmembrane.
