# Supplementary material for: 4-Heteroaryl Substituted Amino-3,5-Dicyanopyridines as New Adenosine Receptor Ligands: Novel Insights on Structure-Activity Relationships and Perspectives
Source: Pharmaceuticals (Basel). 2022 Apr 14;15(4):478. doi: 10.3390/ph15040478 (PMC9024521; doi:10.3390/ph15040478)
Supplement: Supplementary file 1 [file pharmaceuticals-15-00478-s001.zip › pharmaceuticals-1641343-supplementary.pdf]

# Supplementary Material

# 4-Heteroaryl Substituted Amino-3,5-Dicyanopyridines as New Adenosine Receptor Ligands: Novel Insights on Structure-Activity Relationships and Perspectives

Daniela Catarzi [1,*], Flavia Varano [1], Erica Vigiani [1], Sara Calenda [1], Fabrizio Melani [1], Katia Varani [2], Fabrizio Vincenzi [2], Silvia Pasquini [2], Natascia Mennini [3], Giulia Nerli [3], Diego Dal Ben [4], Rosaria Volpini [4] and Vittoria Colotta [1]

**Contents**

***$^{1}$H NMR study of compound 10*** — ***Figures S1-S6***

***Combustion Analysis data of the newly synthesized compounds*** — ***Table S1***

***$^1$H NMR study of compound 10***

After addition of deuterated water ($D_2O$) to the sample of purified compound **10** (as reference), its $^1$H-NMR spectrum underwent a slightly change but intriguing. In fact, the signals of the two imidazole proton at 4,5 (6.85 and 7.07 ppm) did not change chemical shift but widened slightly at the base (Spectrum S3, compare with S2). Obviously, the OH and NH proton signals (12.14 and 11.82 ppm, respectively) disappeared from the spectrum since they exchanged with deuterium. The deuterium of the ND group formed a stronger intramolecular interaction with the pyridine nitrogen with respect to the NH hydrogen [57]. After 24 hours, the signals of the imidazole protons tended to collapse, reducing in intensity and widening in the range 6.8-7.2 ppm (Spectrum S4). After 48 hours, the signal was a broad singlet at 6.96 ppm (Spectrum S5). A week after, a little broadened singlet at 6.98 ppm (Spectrum S6) was present that in a period of about 4 week got closer and closer to 7.02 ppm as in the spectrum of compound **10** before purification (Spectrum S1). This behavior might indicate the disruption of the intramolecular hydrogen bond due to a slow perturbative effect of small quantity of $H_2O$. In fact, compound **10** via the NH imidazole function might form a hydrogen bond with $H_2O$ that is hypothetically more stable than the intramolecular one. However, deuterated solvents (DMSO-$d_6$) used for NMR spectroscopy were almost anhydrous. Thus, a drop of $H_2O$ was added to the sample of **10** in DMSO-$d_6$. By recording the $^1$H-NMR spectrum in these conditions, the imidazole proton signals at 6.85 and 7.07 ppm that were present in pure DMSO-$d_6$ (Spectrum S2) immediately collapse into a single signal at 7.02 ppm that integrated 2, indicating the equivalence of the two imidazole protons at 4,5 positions. This behavior was the same as that observed after 1 weeks by adding a single drop of $D_2O$ to the sample (Spectrum S6). Thus, it could be hypothesized that in pure DMSO-$d_6$, or even in the presence of trace of water, the H-bond was formed. However, this bond can be broken when the amount of water in the environment tends to increase. The hypothesis of the intramolecular H-bond formation between the pyridine nitrogen and the imidazole NH were supported by the lower chemical shift of the imidazole H-5 signal occurring when the vicinal nitrogen engaged hydrogen bond with a proton donor [58]. In fact, when the formation of an intramolecular H-bond was hypothesized, in the $^1$H-NMR spectrum of **10**, the imidazole H-5 signal fell at 6.85 ppm; in contrast, in absence of intramolecular H-bond, the same appeared at 7.02 ppm. Moreover, referring to compound **10**, interaction of $H_2O$ with the NH imidazole proton, resulting in the disruption of the intramolecular H-bond, caused a downfield shift of the NH proton signal (from 11.82 to 12.4 ppm) [59].

Figures S1-S6 below show the different $^1$H NMR spectra registered in DMSO-$d_6$ for compound **10**.

**Figures S1** reports the $^{1}$H NMR spectrum of compound **10** before purification (crude product).

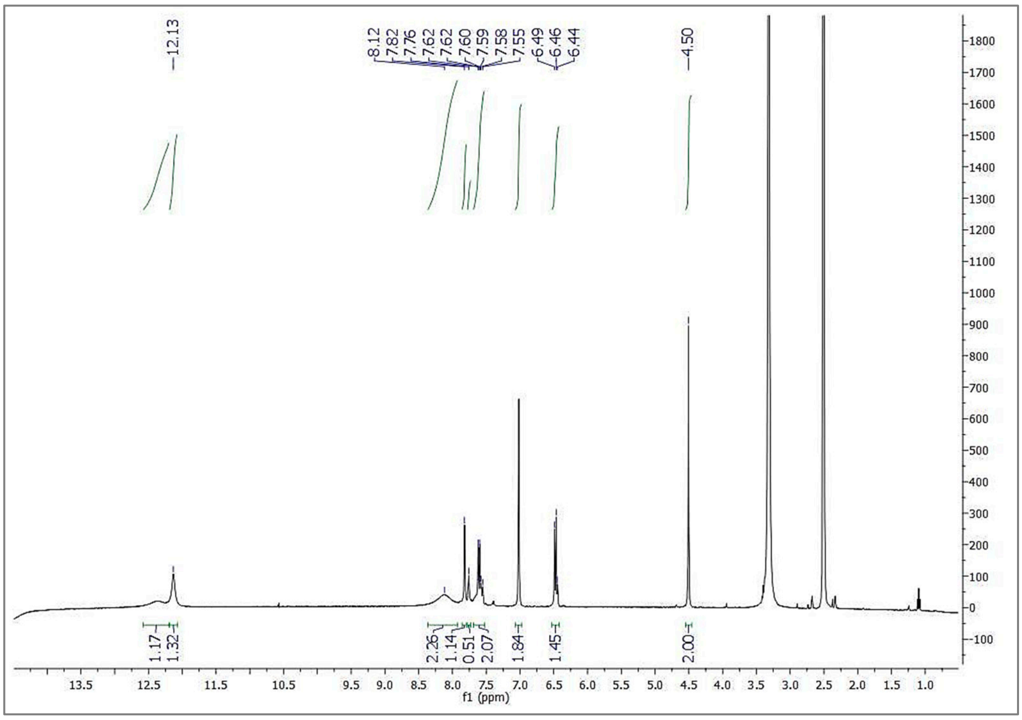


**Figure S2**. $^{1}$H NMR spectrum of compound **10** in DMSO-$d_6$ after purification by crystallization.

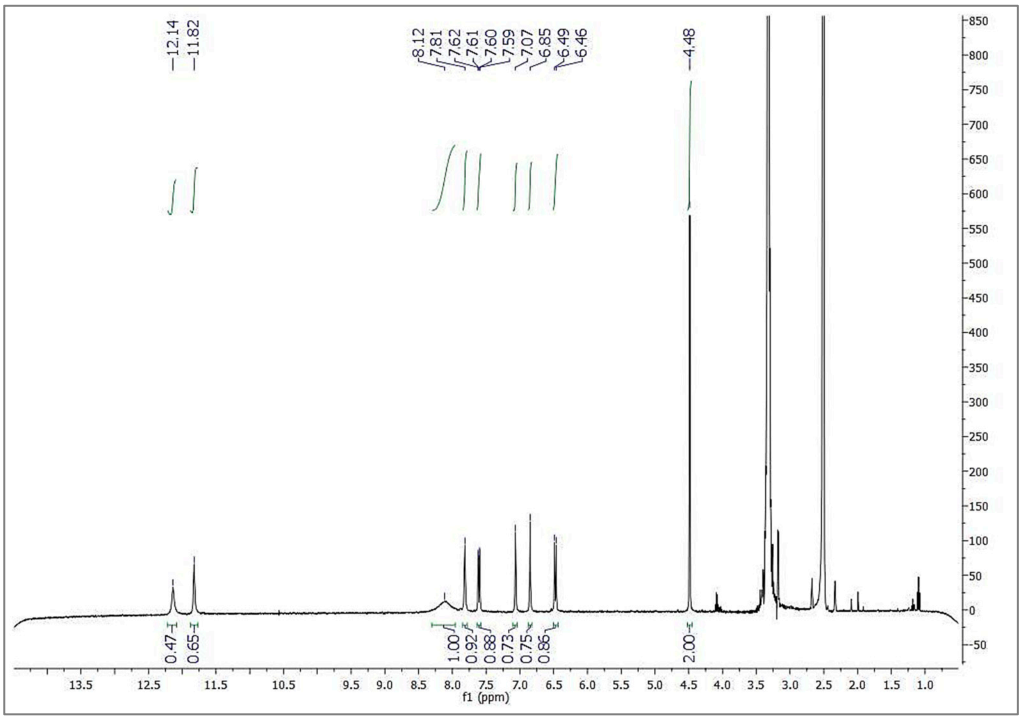

**Figure S3.** $^{1}$H NMR spectrum of purified compound **10** in DMSO-d$_6$ + $D_2O$ (a drop).

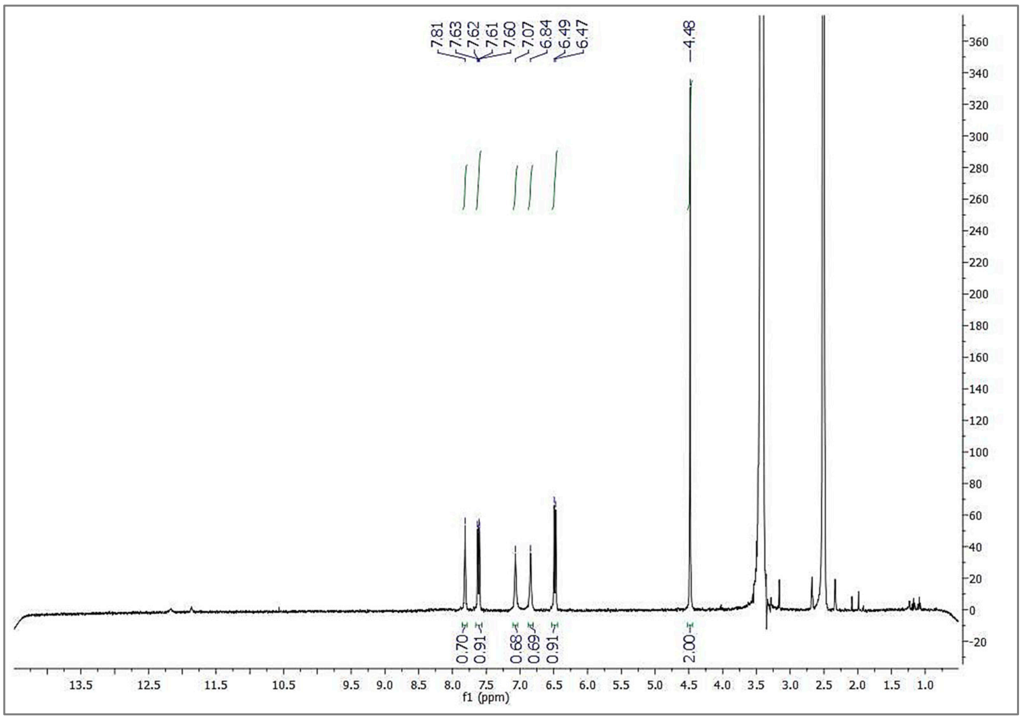


**Figure S4.** $^{1}$H NMR spectrum of purified compound **10** in DMSO-d$_6$ 24 h after adding a drop of $D_2O$.

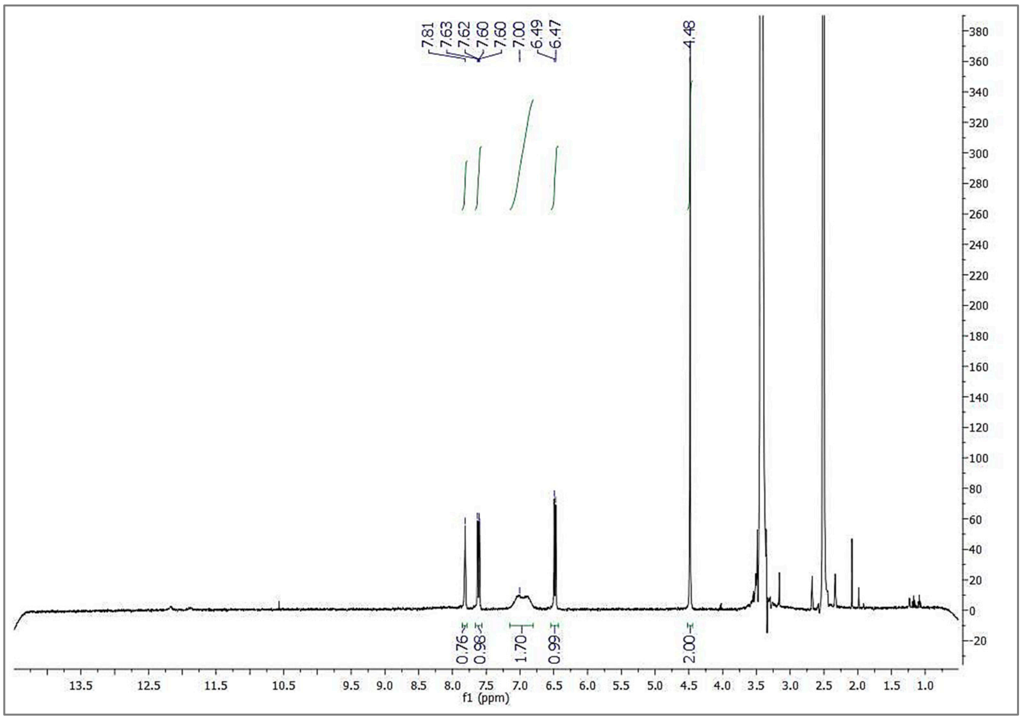

**Figure S5**. $^{1}$H NMR spectrum of purified compound **10** in DMSO-d$_6$ 48 h after adding a drop of $D_2O$.

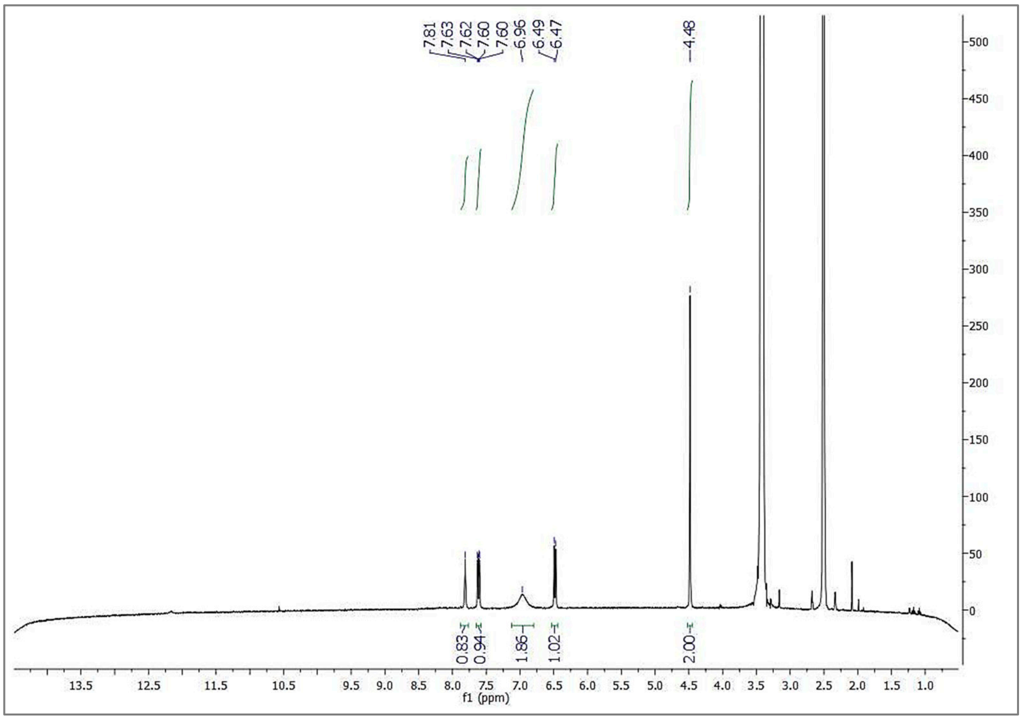


**Figure S6.** $^{1}$H NMR spectrum of purified compound **10** in DMSO-d$_6$ one week after adding a drop of $D_2O$.

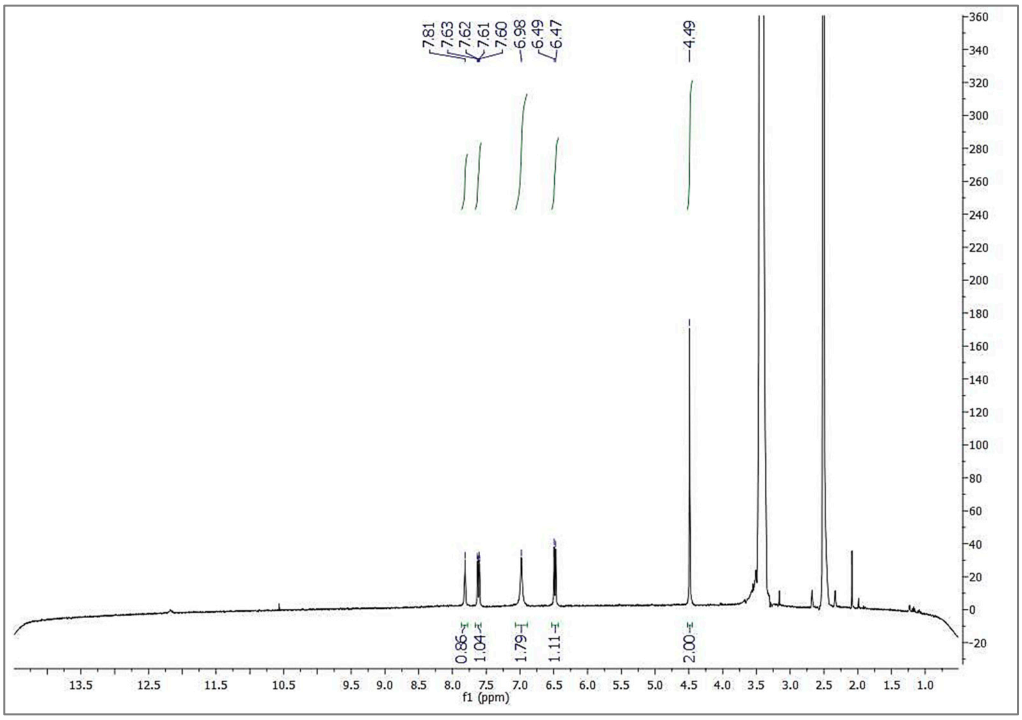

***Table S1.*** *Combustion Analysis data of the newly synthesized compounds* ***1-21, 23, 24, 30, 31, 33, 34, 38, 40, 41, 44-50.***

| compd | Formula | C | H | N |
|---|---|---|---|---|
| | | calcd-found | calcd-found | calcd-found |
| **1** | $C_{15}H_{10}N_6OS$ | 55.89-55.65 | 3.13-3.26 | 26.07-26.35 |
| **1**•HBr | $C_{15}H_{11}BrN_6OS$ | 44.68-44.89 | 2.75-2.59 | 20.84-20.56 |
| **2** | $C_{16}H_{13}BrN_6O_2S$ | 44.35-44.70 | 3.02-3.23 | 19.40-19.17 |
| **3** | $C_{16}H_{12}N_6OS$ | 57.13-57.28 | 3.60-3.44 | 24.98-24.71 |
| **4** | $C_{15}H_{10}N_6OS$ | 55.89-55.57 | 3.13-3.41 | 26.07-26.40 |
| **5** | $C_{15}H_{10}N_6S_2$ | 53.24-52.98 | 2.98-2.77 | 24.83-25.15 |
| **6** | $C_{15}H_{10}N_6S_2$ | 53.24-53.01 | 2.98-2.68 | 24.83-25.02 |
| **7** | $C_{16}H_{11}N_7S$ | 57.64-57.91 | 3.33-3.05 | 29.41-29.08 |
| **8** | $C_{16}H_{11}N_7S$ | 57.64-57.89 | 3.33-3.49 | 29.41-29.29 |
| **9** | $C_{17}H_{13}N_7OS$ | 56.19-55.85 | 3.61-3.75 | 26.98-27.22 |
| **10** | $C_{16}H_{11}N_7OS$ | 55.01-54.77 | 3.17-2.93 | 28.06-28.35 |
| **11** | $C_{15}H_{11}ClN_6OS$ | 50.21-50.50 | 3.09-2.87 | 23.42-23.31 |
| **12** | $C_{15}H_{11}N_7O$ | 59.01-59.28 | 3.63-3.54 | 32.12-31.87 |
| **13** | $C_{16}H_{13}N_7O$ | 60.18-60.33 | 4.10-3.93 | 30.70-30.54 |
| **14** | $C_{17}H_{13}N_7O_2S$ | 53.82-53.58 | 3.45-3.61 | 25.84-26.03 |
| **15** | $C_{17}H_{13}N_7O_2S$ | 53.82-54.10 | 3.45-3.38 | 25.84-25.67 |
| **16** | $C_{18}H_{15}N_7O_2S$ | 54.95-55.22 | 3.84-3.68 | 24.92-24.81 |
| **17** | $C_{21}H_{16}N_8O_2S_2$ | 52.93-52.71 | 3.38-3.56 | 23.51-23.82 |
| **18** | $C_{20}H_{18}N_6OS$ | 61.52-61.28 | 4.65-4.82 | 21.52-21.69 |
| **19** | $C_{18}H_{14}N_6OS$ | 59.65-59.90 | 3.89-3.77 | 23.19-22.94 |
| **20** | $C_{19}H_{16}N_6OS$ | 60.62-60.33 | 4.28-4.36 | 22.33-22.05 |
| **21** | $C_{17}H_{12}N_6O_2S$ | 56.04-56.34 | 3.32-3.51 | 23.06-22.86 |
| **23** | $C_{18}H_{12}N_4O_2S$ | 62.06-61.88 | 3.47-3.61 | 16.08-15.79 |
| **24** | $C_{18}H_{12}N_4OS$ | 65.04-65.31 | 3.64-3.49 | 16.86-16.62 |
| **30** | $C_{19}H_{13}N_5OS$ | 63.49-63.75 | 3.65-3.81 | 19.49-19.15 |
| **31** | $C_{18}H_{11}N_5OS$ | 62.60-62.33 | 3.21-3.35 | 20.28-20.42 |
| **33** | $C_{12}H_8N_4O_2S$ | 52.93-53.29 | 2.96-2.73 | 20.58-20.41 |
| **34** | $C_{12}H_8N_4OS$ | 56.24-56.55 | 3.15-2.99 | 21.86-21.68 |
| **38** | $C_{12}H_7N_5S$ | 56.90-56.65 | 2.79-3.00 | 27.65-27.51 |
| **40** | $C_{13}H_9N_5OS$ | 55.11-55.40 | 3.20-3.33 | 24.72-24.49 |
| **41** | $C_{12}H_7N_5OS$ | 53.52-53.28 | 2.62-2.76 | 26.01-26.32 |
| **44** | $C_{17}H_8ClN_3OS$ | 60.45-60.31 | 2.39-2.56 | 12.44-12.28 |
| **45** | $C_{22}H_{18}N_4OS$ | 68.37-68.12 | 4.69-4.83 | 14.50-14.72 |
| **46** | $C_{20}H_{14}N_4OS$ | 67.02-66.78 | 3.94-4.22 | 15.63-15.77 |
| **47** | $C_{21}H_{16}N_4OS$ | 67.72-68.05 | 4.33-4.09 | 15.04-14.88 |
| **48** | $C_{16}H_{14}N_4OS$ | 61.92-61.76 | 4.55-4.67 | 18.05-17.91 |
| **49** | $C_{14}H_{10}N_4OS$ | 59.56-59.73 | 3.57-3.70 | 19.85-20.03 |
| **50** | $C_{15}H_{12}N_4OS$ | 60.79-61.06 | 4.08-3.94 | 18.91-18.62 |
